# Menopause impacts human brain structure, connectivity, energy metabolism, and amyloid-beta deposition

**DOI:** 10.1038/s41598-021-90084-y

**Published:** 2021-06-09

**Authors:** Lisa Mosconi, Valentina Berti, Jonathan Dyke, Eva Schelbaum, Steven Jett, Lacey Loughlin, Grace Jang, Aneela Rahman, Hollie Hristov, Silky Pahlajani, Randolph Andrews, Dawn Matthews, Orli Etingin, Christine Ganzer, Mony de Leon, Richard Isaacson, Roberta Diaz Brinton

**Affiliations:** 1 Department of Neurology, Weill Cornell Medicine, 420 East 70th, LH-404, New York, NY 10021 USA; 2 Department of Radiology, Weill Cornell Medical College, New York, NY USA; 3 Department of Psychiatry, New York University School of Medicine, New York, NY USA; 4 Department of Nuclear Medicine, University of Florence, Florence, Italy; 5 ADM Diagnostics, Chicago, IL USA; 6 Department of Internal Medicine, Weill Cornell Medical College, New York, NY USA; 7 Hunter-Bellevue School of Nursing, Hunter College, CUNY, New York, NY USA; 8 Departments of Pharmacology and Neurology, College of Medicine, University of Arizona, Tucson, AZ USA

**Keywords:** Cognitive ageing, Biomarkers, Medical research, Neurology, Risk factors

## Abstract

All women undergo the menopause transition (MT), a neuro-endocrinological process that impacts aging trajectories of multiple organ systems including brain. The MT occurs over time and is characterized by clinically defined stages with specific neurological symptoms. Yet, little is known of how this process impacts the human brain. This multi-modality neuroimaging study indicates substantial differences in brain structure, connectivity, and energy metabolism across MT stages (pre-menopause, peri-menopause, and post-menopause). These effects involved brain regions subserving higher-order cognitive processes and were specific to menopausal endocrine aging rather than chronological aging, as determined by comparison to age-matched males. Brain biomarkers largely stabilized post-menopause, and gray matter volume (GMV) recovered in key brain regions for cognitive aging. Notably, GMV recovery and in vivo brain mitochondria ATP production correlated with preservation of cognitive performance post-menopause, suggesting adaptive compensatory processes. In parallel to the adaptive process, amyloid-β deposition was more pronounced in peri-menopausal and post-menopausal women carrying apolipoprotein E-4 (APOE-4) genotype, the major genetic risk factor for late-onset Alzheimer’s disease, relative to genotype-matched males. These data show that human menopause is a dynamic neurological transition that significantly impacts brain structure, connectivity, and metabolic profile during midlife endocrine aging of the female brain.

## Introduction

The menopause transition (MT) is a midlife neuroendocrine aging process specific to females that culminates with reproductive senescence^1^. All women undergo menopause in their lifetime either through the natural endocrine aging process or through medical intervention. The MT occurs in stages characterized by unique endocrine properties that impact aging trajectories of multiple organ systems including brain^1^. While menopause is a reproductive transition state, it is also a neurological transition^1^, as evidence by the fact that many menopausal symptoms are neurological in nature, such as hot flashes, disturbed sleep, mood changes, and forgetfulness^2^.

The MT is orchestrated by gonadal sex steroid hormones, which are known regulators of both reproductive and neural function^1^. During the MT, sex hormones, especially 17β-estradiol, substantially decline in body and brain^3^. Molecular, cellular and systems biology characterization of the MT in brain demonstrate a broad impact of estrogen declines on neural processes via genomic and non-genomic actions^1^, including changes in spinogenesis and synaptogenesis, neuronal number, morphology, glucose metabolic rates, and gene expression^1,3^. Additionally, estrogen depletion has been linked to amyloid-beta (Aβ) plaque accumulation, a hallmark of Alzheimer’s disease (AD), in female animals^4,5^. However, little is known of how the MT impacts the human brain.

Brain imaging analyses conducted in post-menopausal women first reported declines in glucose metabolism and gray matter volume (GMV), which were modulated by menopause hormone treatment (HT)^6,7^, indicating that neuro-endocrine processes retain dynamic properties well into menopause. However, data in women *undergoing* the MT are scarce. We previously reported that, among those with genetic risk factors for AD such as apolipoprotein epsilon-4 genotype (APOE-4)^8^, the MT was associated with lower GMV, hypometabolism, and emerging Aβ deposition^9–11^. It is unknown whether similar changes occur in women without genetic risks for AD, or whether the MT impacts other structural, bioenergetic and cognitive indicators.

Herein, we conducted a multi-modality neuroimaging study of women at different MT stages (pre-, peri-, and post-menopause) to investigate MT’s effects on brain’s gray (GM) and white matter (WM) structure, connectivity, energy metabolism, and Aβ deposition. Results indicate that the MT significantly impacts all these brain biomarkers in regions involved in higher-order cognitive functions. Effects were independent of age and HT use, and were specific to menopausal endocrine aging rather than chronological aging, as determined by comparison with age-matched males. Notably, cognition was preserved post-menopause, which correlated with GMV recovery and brain adenosine triphosphate (ATP) production, highlighting potential compensatory mechanisms. Finally, Aβ deposition was greater in post-menopausal and peri-menopausal women carrying APOE-4 genotype, indicating APOE-4 specific effects on AD risk with onset in the peri-menopause.

## Results

### Participants

A total of 182 cognitively intact 40–65 year-old women were enrolled. Nine participants were excluded due to neuropathological conditions encountered in the MR images (n = 7; e.g. neoplastic condition or aneurysm), or due to artifacts (n = 2). Three participants with incomplete clinical data and 9 with unclear menopause status were also excluded. Our final sample consisted of 161 women, including 30 pre-menopausal (PRE), 57 peri-menopausal (PERI), and 74 post-menopausal (POST) participants.

Participants’ characteristics are found in Table 1. There were no group differences in demographic and clinical measures except for an age difference between PRE and POST groups. As described in “Methods”, our procedures to address age effects included (i) adding age as a confounder in all analyses, and (ii) comparing each MT group to an age-matched male group: consisting of 30 males age-matched to the PRE group (MALE_PRE_), 50 age-matched to the PERI group (MALE_PERI_), and 45 age-matched to the POST group (MALE_POST_) (Supplementary Table S1). Forty-two percent of participants were APOE-4 positive, with comparable distributions between groups.

**Table 1:** Participants’ characteristics by menopause status.

	Pre-menopausal group	Peri-menopausal group	Post-menopausal group
**Clinical and demographic measures**			
N	30	57	74
Age, years, range	44 (4), 40–53	50 (4), 41–60	57 (4)*, 46–65
Education, years	17 (3)	17 (2)	17 (2)
Ethnicity, % White	80	77	89
MMSE score	29 (1)	29 (1)	29 (1)
APOE ε4 carriers, % positive	43	33	48
Hysterectomy status, % positive	0	6	19
Menopausal hormonal therapy			
*% Current users*	0	13	32
*% Past users*	0	2	4
**Cognitive measures**			
Global cognition, mean (SE)	− 0.11 (0.17)	− 0.02 (0.09)	0.06 (0.11)
*Adjusted by APOE-4 status*	− 0.09 (0.17)	− 0.03 (0.09)	0.06 (0.11)
Memory, mean (SE)	0.05 (0.20)	0.06 (0.11)	0.02 (0.13)
*Adjusted by APOE-4 status*	0.07 (0.21)	0.04 (0.11)	0.02 (0.13)

Clinical measures are means (SD), unless otherwise specified. Cognitive measures are age and education-adjusted means (SE).

*Different from PRE, *p* < 0.05.

### Biomarker results

We examined a panel of brain biomarkers examining:

- Structure: GMV and white matter volume (WMV) via Magnetic Resonance Imaging (MRI), and fractional anisotropy (FA, an index of WM integrity and structural connectivity^12^) via MRI-Diffusion Tensor Imaging (DTI);
- Energy metabolism: glucose metabolism (CMRglc) via ^18^F-fluorodeoxy-2-glucose (FDG) Positron Emission tomography (PET), cerebral blood flow (CBF) via Arterial Spin Labeling (ASL), and ATP production via ^31^Phosphorus-Magnetic Resonance Spectroscopy (^31^P-MRS);
- Aβ deposition via ^11^C-Pittsburgh compound B (PiB) PET.

Unless otherwise specified, all below voxel-based results are significant at *p* < 0.05, cluster-level corrected for family-wise type error (FWE), adjusted for age, APOE-4 status, and modality-specific confounders.

### Biomarker differences between MT groups

#### Structural biomarkers

##### GMV

**Figure 1:**
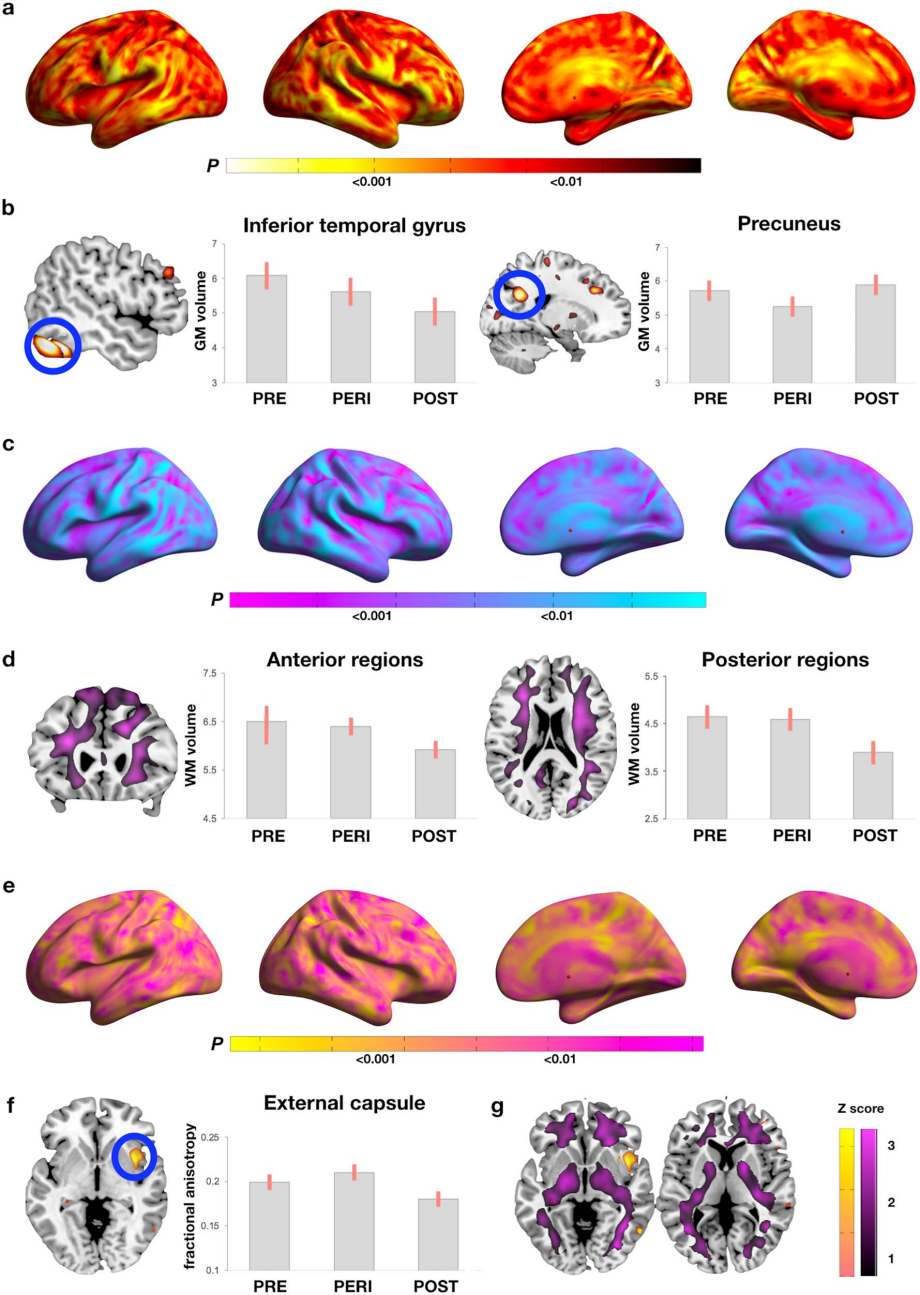
Structural biomarker differences between menopausal groups. (**a**) Surface maps of regional GMV differences. (**b**) MRI slice overlays and plots representing GMV in temporal and precuneus clusters. (**c**) Surface maps of regional WMV differences. (**d**) MRI slice overlays and plots representing WMV in anterior and posterior areas averaged between hemispheres. (**e**) Surface maps of regional FA differences. (**f**) MRI slice overlays and plots representing FA in external capsule. (**g)** Slice overlays depicting lack of overlap between WMV (purple) and FA (yellow) effects. In (**a**, **c**, **e**), SPMs are represented on modality-specific color-coded scales with corresponding P values. In (**g**), Z scores are reported in lieu of *p* values to enable multi-modality comparison. In (**b**, **d**), values are mean (SE). Results are adjusted by age and total intracranial volume. *Abbreviations* FA, fractional anisotropy; GMV, gray matter volume; PERI, peri-menopause; POST, post-menopause; PRE, pre-menopause; SPMs, statistical parametric maps; WMV, white matter volume.

MT stage effects were observed in inferior temporal gyrus, precuneus, and fusiform gyrus of the right hemisphere (Fig. 1a). On post-hoc analysis, the temporal cluster reflected lower GMV in the POST group compared to the PRE group, while the precuneus and fusiform clusters reflected lower GMV in the PERI group compared to the POST group (Fig. 1b, and Supplementary Table S2).

##### WMV

MT stage effects were observed in anterior and posterior corona radiata, which reflected lower WMV in POST versus PRE and PERI groups (Fig. 1c, d, and Supplementary Table S2).

##### FA

The only cluster showing MT stage effects that survived correction for multiple comparisons was the right external capsule. In this cluster, the POST group had lower FA than PERI (cluster extent 71 voxels, *x, y, z* = 33, − 9, − 6, Z = 3.81, *p* = 0.017; Fig. 1e, f). There was no anatomical overlap between FA and WMV maps, as shown in Fig. 1g.

#### Metabolic biomarkers

##### CMRglc

**Figure 2:**
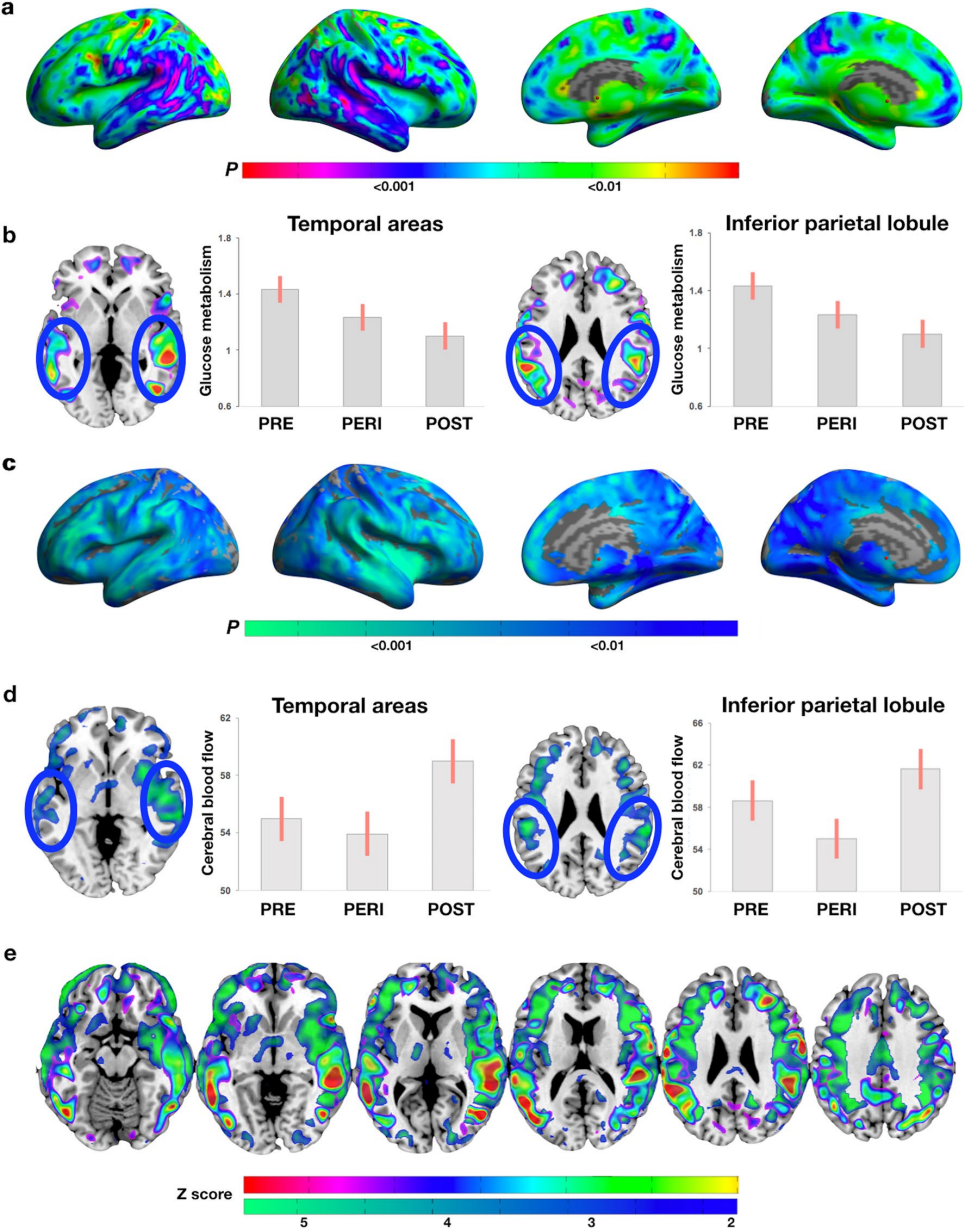
Metabolic biomarker differences between menopausal groups. (**a**) Surface maps of regional CMRglc differences. (**b)** MRI slice overlays and plots representing CMRglc in temporal and parietal regions. (**c**) Surface maps of regional CBF differences. (**d**) MRI slice overlays and plots representing CBF extracted in temporal and parietal clusters. (**e**) Slice overlays depicting the overlap between CMRglc (green to red scale) and CBF (blue to green scale) effects. (**f**) In (**a**, **c**), SPMs are represented on modality-specific color-coded scales with corresponding *p* values. In (**e**), Z scores are reported to enable multi-modality comparisons. In (**b**, **d**), values are mean (SE). Results are adjusted by age and global activity. *Abbreviations* CBF, cerebral blood flow; CMRglc, cerebral glucose metabolism; PERI, peri-menopause; POST, post-menopause; PRE, pre-menopause; SPMs, statistical parametric maps.

MT stage effects were observed in supramarginal gyri, middle and inferior temporal gyri (Fig. 2a). On post-hoc analysis, all clusters reflected lower CMRglc in the POST group compared to the PRE and PERI groups, and lower CMRglc in right middle temporal gyrus of the PERI compared to the PRE group (Fig. 2b, and Supplementary Table S3).

##### CBF

MT stage effects were observed in supramarginal gyrus, middle and superior temporal gyrus, superior and inferior frontal gyrus of both hemispheres, which on post-hoc analysis, reflected higher CBF in POST versus PERI (Fig. 2c, d, and Supplementary Table S3).

Figure 2e displays the regional overlap of CMRglc and CBF effects.

##### ATP production

We examined regional ATP to phosphocreatine (PCr) ratios in parieto-temporal regions showing MT-stage effects on CMRglc and CBF (Table 2). Multivariate general linear models (mGLM) showed higher ATP/PCr in temporal regions (*p*’s ≤ 0.047) and borderline higher ATP/PCr in parietal regions (*p* < 0.12) of POST versus PRE groups. Results remained significant adjusting by APOE-4 status (Table 2).

**Table 2:** Region-of-Interest (ROI) measures by menopausal stage group.

	PRE	PERI	POST
**ATP/PCr measures**			
Superior temporal gyrus	1.11 (0.05)	1.18 (0.03)	1.26 (0.03)*
*Adjusted by APOE status*	1.14 (0.04)	1.18 (0.03)	1.24 (0.02)*
Middle temporal gyrus	1.09 (0.04)	1.13 (0.03)	1.20 (0.02)*
*Adjusted by APOE status*	1.10 (0.03)	1.14 (0.02)	1.19 (0.02)*
Inferior temporal gyrus	1.07 (0.04)	1.11 (0.02)	1.13 (0.02)*
*Adjusted by APOE status*	1.07 (0.03)	1.10 (0.02)	1.14 (0.02)*
Inferior parietal lobule	0.77 (0.06)	0.76 (0.05)	0.83 (0.04)
*Adjusted by APOE status*	0.78 (0.04)	0.75 (0.03)	0.83 (0.03)*
**Amyloid-β load**			
AD-mask SUVR	1.01 (0.12)	1.20 (0.06)	1.27 (0.07)
*Adjusted by APOE status*	*1.00 (0.09)*	*1.21 (0.06)*	1.29 (0.06)*
AD-mask SUVR by APOE-4 status			
APOE-4−	0.94 (0.12)	1.14 (0.07)	1.32 (0.07)*
APOE-4+	1.07 (0.12)	1.32 (0.11)*	1.25 (0.08)*

APOE-4−, APOE-4 non-carriers; APOE-4+, APOE-4 carriers; PERI, peri-menopausal group; POST, post-menopausal group; PRE, pre-menopausal group; SUVR, standardized uptake value ratio to cerebellar gray matter PiB uptake.

Values are means (SE), unless otherwise specified.

*Different from PRE, *p* < 0.05.

#### Amyloid-β load

Results are reported in Table 2. Adjusting by age and cerebellar uptake, there were no significant differences in PiB uptake between MT groups. However, adding APOE-4 status as a covariate enhanced group differences, resulting in higher PiB uptake in POST versus PRE (27%, *p* = 0.006), and PERI versus PRE groups (18%, *p* = 0.056). PiB differences between PERI and PRE groups were more pronounced among APOE-4 carriers (Table 2).

### Comparisons to age-matched males

To determine whether MT-stage effects on biomarkers were specific for menopause endocrine aging versus chronological aging, we compared each MT group to an age-matched male group. We first tested for group biomarker differences within the brain regions showing the MT group-specific effects reported above. We then tested for group differences in the entire search volume.

#### Structural biomarkers

##### GMV

**Figure 3:**
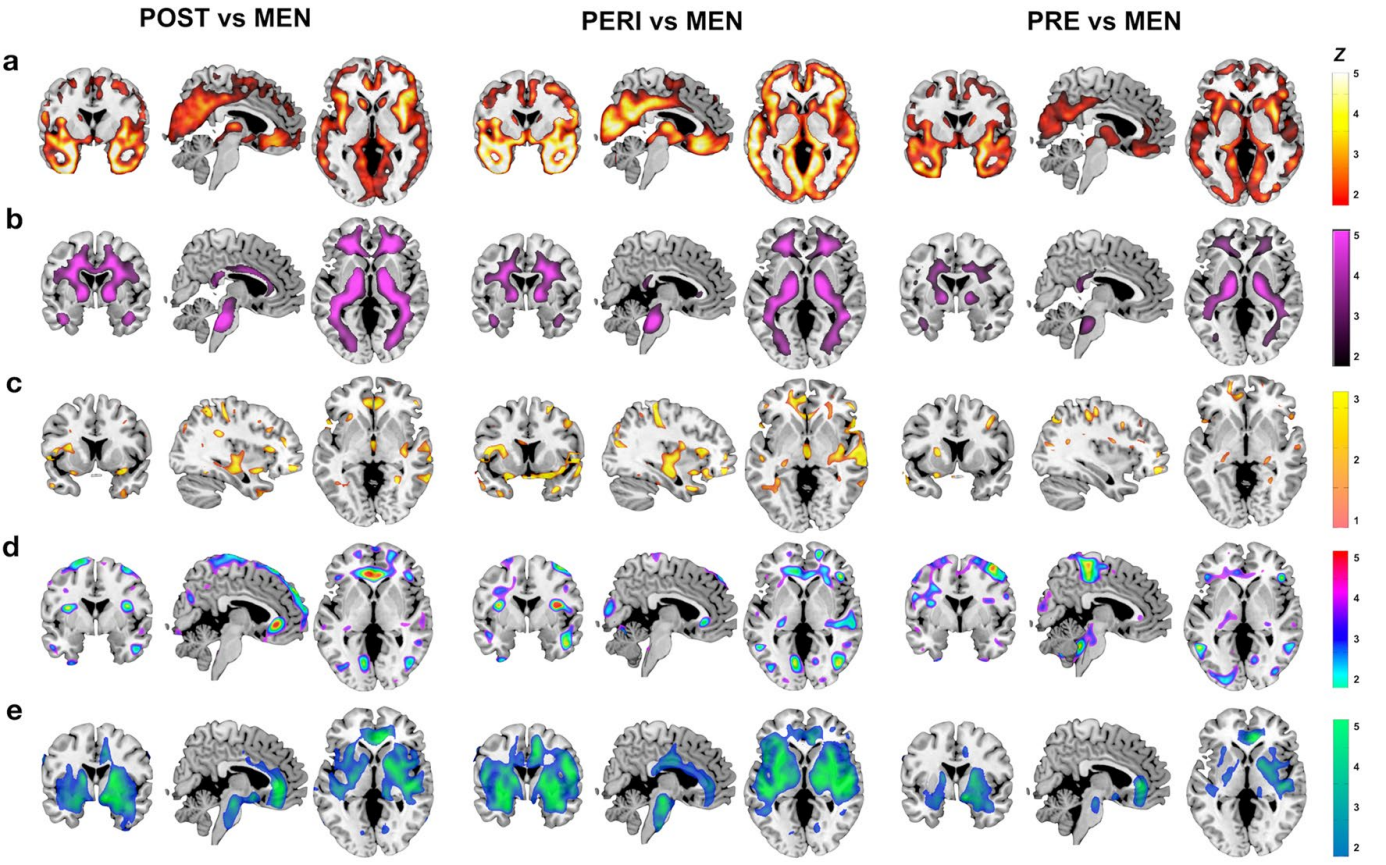
MRI slice overlays displaying biomarker differences between each MT group and males in the corresponding age ranges: (**a**) Lower GMV in (left) POST, (middle) PERI, and (right) PRE groups versus males. (**b**) Lower WMV in (left) POST, (middle) PERI, and (right) PRE groups versus males. (**c**) Higher FA in (left) POST, and (middle) PERI versus males; (right) no differences between PRE and males. (**d**) Lower CMRglc in (left) POST, (middle) PERI, and (right) PRE groups versus males. (**e**) Higher CBF in (left) POST, (middle) PERI, and (right) PRE groups versus males. (**f**) SPMs are represented on modality-specific color-coded scales with corresponding Z scores to enable multi-modality comparisons. *Abbreviations* See legend to Figs. 1 and 2.

Results are presented in Fig. 3a, and Supplementary Table S4. Examination of brain regions showing MT-stage effects indicated lower GMV in inferior temporal gyrus of the POST group compared to MALE_POST_, and lower GMV in precuneus and fusiform gyrus of the PERI group compared to MALE_PERI_. Across the entire search volume, both POST and PERI groups exhibited additional widespread areas of lower GMV compared to males, involving middle and medial temporal (MTL) regions, superior, middle, and orbital frontal gyrus, anterior cingulate (ACC), insula, and putamen. The PRE group showed lower GMV versus MALE_PRE_ in MTL, frontal gyrus, putamen, and inferior and middle temporal cortex.

##### WMV

Results are presented in Fig. 3b, and Supplementary Table S5. In examination of brain regions exhibiting MT-stage effects, lower WMV in corona radiata of the POST group compared to MALE_POST_ was evident. In the entire search volume, the POST group exhibited additional areas of lower WMV in several tracts including superior longitudinal fasciculus (SLF), posterior thalamic radiation, and cortico-spinal tracts. The PERI group exhibited a similar pattern of lower WMV compared to MALE_PERI_, and the PRE group showed lower WMV in internal capsule compared to MALE_PRE_.

##### FA

Results are presented in Fig. 3c, and Supplementary Table S6. Examination of brain regions showing MT-stage effects revealed higher FA in corona radiata and fornix in the POST group compared to MALE_POST_. There were no other regions showing FA differences for POST versus MALE_POST._ The PERI group exhibited higher FA in corona radiata, fornix, external capsule and uncinate fasciculus; and lower FA in SLF and posterior thalamic radiation compared to MALE_PERI_. The PRE group showed higher FA in corona radiata, and lower FA in SLF, compared to MALE_PRE_.

#### Metabolic biomarkers

##### CMRglc

Results are presented in Fig. 3d, and Supplementary Table S7. Examination of brain regions showing MT-stage effects showed lower CMRglc in temporo-parietal areas of the POST group compared to MALE_POST_. In the entire search volume, the POST group exhibited additional areas of hypometabolism in superior and middle frontal cortex compared to males. The PERI group showed lower CMRglc in frontal cortex and insula versus MALE_PERI_, and the PRE group showed lower CMRglc in left supramarginal gyrus versus MALE_PRE_.

##### CBF

Results are presented in Fig. 3e, and Supplementary Table S8. Examination of brain regions exhibiting MT-stage effects showed elevated CBF in frontal regions of POST versus MALE_POST_. In the entire search volume, the POST group also exhibited lower CBF in ACC, MTL, and basal ganglia compared to males. Likewise, PERI and PRE groups exhibited areas of higher and lower CBF compared to males, chiefly lower CBF in ACC, medial and superior temporal gyrus, and basal ganglia, and higher CBF in parietal lobe.

##### ATP production

We investigated ATP/PCr using two separate mGLMs: model 1 included regions with higher CBF in POST versus MALE_POST_ (ACC, insula and temporo-parietal regions); model 2 included regions with lower CMRglc and CBF in POST versus MALE_POST_ (frontal areas). Results are presented in Table 3. With and without adjustment by APOE-4 status, the POST group had higher ATP/PCr than MALE_POST_ in model 1 (*p* ≤ 0.003) and borderline higher ATP/PCr in model 2 (*p* ≤ 0.082), and the PERI group had higher ATP/PCr than MALE_PERI_ in model 2 (*p* ≤ 0.049). There were no differences between PRE and MALE_PRE_.

**Table 3:** Regions-of-interest (ROI) measures by age-matched male group comparisons.

	PRE	MALE_PRE_	PERI	MALE_PERI_	POST	MALE_POST_
**ATP/PCr measures**						
Anterior cingulate cortex	1.06 (0.07)	1.11 (0.07)	1.10 (0.04)	1.12 (0.05)	1.12 (0.04)	1.12 (0.05)
*Adjusted by APOE-4 status*	1.05 (0.07)	1.12 (0.07)	1.11 (0.04)	1.10 (0.05)	1.11 (0.04)	1.13 (0.05)
Insula	1.21 (0.06)	1.29 (0.07)	1.24 (0.04)	1.24 (0.04)	1.26 (0.03)*	1.21 (0.05)
*Adjusted by APOE-4 status*	1.20 (0.06)	1.30 (0.06)	1.24 (0.04)	1.23 (0.04)	1.27 (0.03)*	1.22 (0.05)
Inferior parietal lobule	0.78 (0.07)	0.85 (0.08)	0.76 (0.04)	0.84 (0.04)	0.83 (0.03)	0.86 (0.04)
*Adjusted by APOE-4 status*	0.77 (0.08)	0.86 (0.08)	0.76 (0.04)	0.84 (0.04)	0.83 (0.03)	0.86 (0.04)
Middle temporal gyrus	1.10 (0.04)	1.17 (0.05)	1.15 (0.02)	1.12 (0.3)	1.19 (0.02)*	1.10 (0.03)
*Adjusted by APOE-4 status*	1.10 (0.04)	1.18 (0.04)	1.15 (0.02)	1.12 (0.3)	1.19 (0.02)*	1.10 (0.03)
Medial temporal lobe	1.16 (0.05)	1.24 (0.05)	1.22 (0.03)	1.20 (0.04)	1.21 (0.03)	1.19 (0.04)
*Adjusted by APOE-4 status*	1.15 (0.05)	1.26 (0.05)	1.22 (0.03)	1.20 (0.04)	1.22 (0.03)	1.19 (0.04)
Medial frontal gyrus	1.05 (0.06)	0.99 (0.06)	1.04 (0.03)	0.98 (0.04)	1.07 (0.03)*	0.95 (0.04)
*Adjusted by APOE-4 status*	1.04 (0.06)	1.01 (0.06)	1.05 (0.03)	0.97 (0.04)	1.07 (0.03)*	0.96 (0.04)
Superior frontal gyrus	0.95 (0.05)	0.86 (0.06)	0.94 (0.03)*	0.84 (0.03)	0.96 (0.03)*	0.83 (0.04)
*Adjusted by APOE-4 status*	0.94 (0.05)	0.87 (0.05)	0.95 (0.03)*	0.84 (0.04)	0.96 (0.03)*	0.84 (0.04)
**Amyloid-β load**						
AD-mask SUVR	1.01 (0.05)	0.92 (0.05)	1.20 (0.05)*	0.92 (0.06)	1.29 (0.05)*	0.93 (0.06)
*Adjusted by APOE-4 status*	1.01 (0.05)	0.92 (0.05)	1.21 (0.05)*	0.91 (0.06)	1.29 (0.05)*	0.93 (0.06)
AD-mask SUVR by APOE-4 status						
APOE-4−	0.94 (0.07)	0.86 (0.07)	1.14 (0.06)	0.88 (0.08)	1.32 (0.07)*	0.93 (0.09)
APOE-4+	1.07 (0.07)	0.96 (0.08)	1.32 (0.09)*	0.93 (0.10)	1.25 (0.05)*	0.95 (0.10)

MALE_PRE_, males age-matched to PRE; MALE_PERI_, males age-matched to PERI; MALE_POST_, males age-matched to POST; PERI, peri-menopausal group; POST, post-menopausal group; PRE, pre-menopausal group.

Values are means (SE).

*Different from age-matched males, *p* < 0.05.

#### Amyloid-β load

POST and PERI groups exhibited higher PiB uptake in AD-mask than MALE_POST_ and MALE_PERI_, respectively (*p*’s ≤ 0.001), while no differences were found between PRE and MALE_PRE_ (Table 3). These effects remained unchanged including APOE-4 status as a covariate, which enhanced differences between PERI and MALE_PERI_ APOE-4 carriers (*p* = 0.020). PiB differences between PERI and MALE_PERI_ were more pronounced among APOE-4 carriers (Table 3).

### Biomarker changes post-menopause

To examine whether biomarker effects were persistent post-menopause, and to test their specificity for menopause endocrine aging versus chronological aging, we performed 2-year follow-up volumetric MRI and FDG-PET scans in 17 POST and 12 MALE_POST_ (Supplementary Table S9). We first tested for changes in biomarkers within the brain regions showing POST-specific effects, and secondly, in the entire search volume.

**Figure 4:**
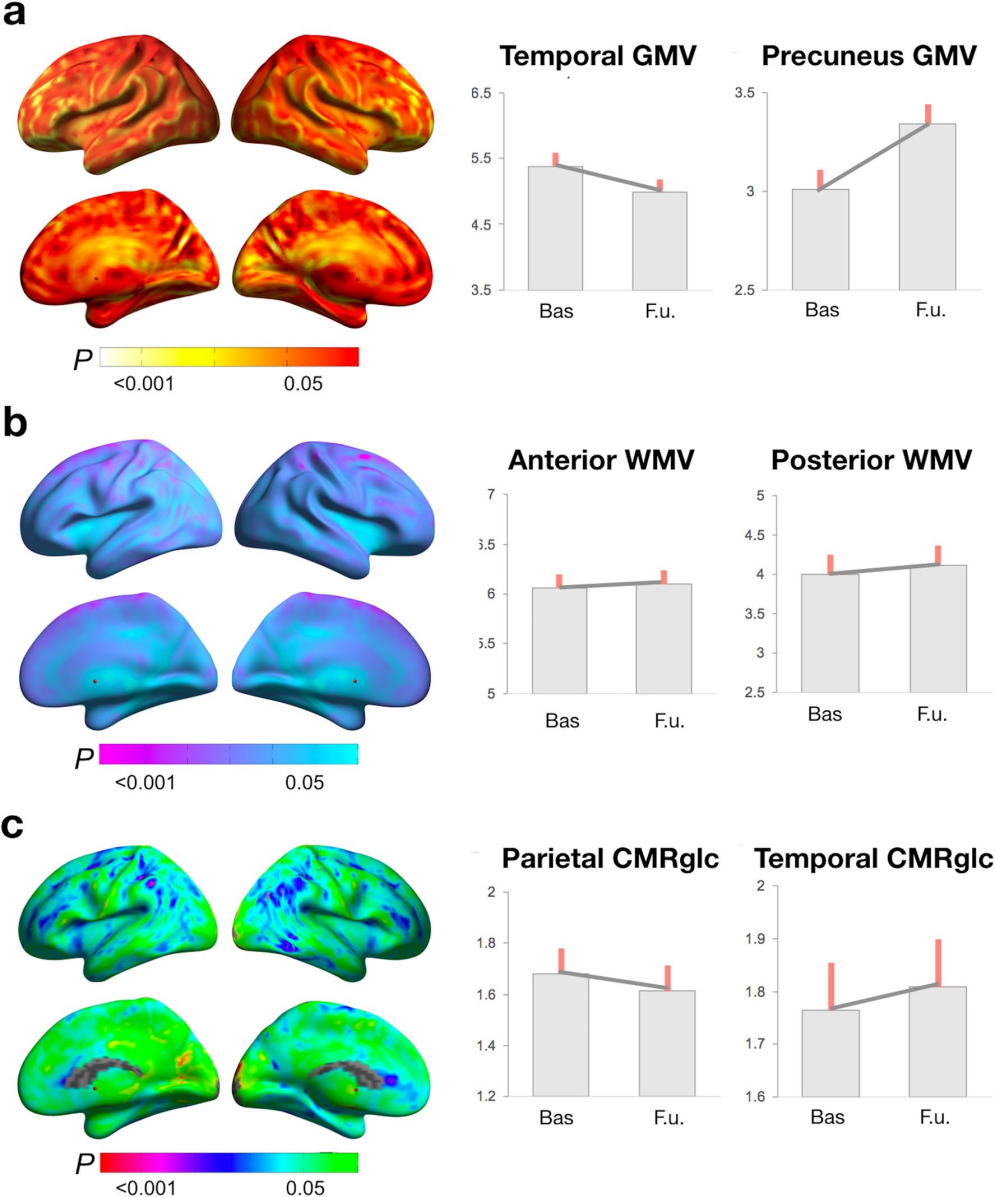
Longitudinal biomarker changes post-menopause. (**a)** Surface maps of GMV change. (**b**) Surface maps of WMV change. (**c**) Surface maps of CMRglc change. (**d**) Plots representing mean (SE) biomarker levels at baseline (POST) versus 2-year follow-up (POST + 2) in the subset of POST participants with longitudinal brain scans. Surface maps are represented on modality-specific color-coded scales with corresponding *p* values. See legends to Figs. 1 and 2.

Examination of brain regions exhibiting POST-stage effects on GMV indicated no GMV changes in inferior temporal and fusiform gyri, and GMV increases in the precuneus of the POST group (Fig. 4a, d; and Supplementary Table S10). There were no GMV changes in precuneus of the MALE_POST_ group, suggesting POST-specific GMV recovery. In the entire search volume, both POST and MALE_POST_ groups showed GMV declines in right frontal gyrus, which did not differ between the groups (Supplementary Table S10).

No significant WMV changes were observed in the POST group, or in comparison to MALE_POST_ (Fig. 4b, d).

Examination of temporo-parietal regions that were hypometabolic in the POST group versus PRE and PERI groups showed CMRglc declines in left inferior parietal lobule and not in other areas of the POST group (Fig. 4c, d; Supplementary Table S11). Parietal CMRglc declines were significant compared to MALE_POST_. In the entire search volume, there were no further longitudinal group differences.

### Summary of biomarker findings

**Figure 5:**
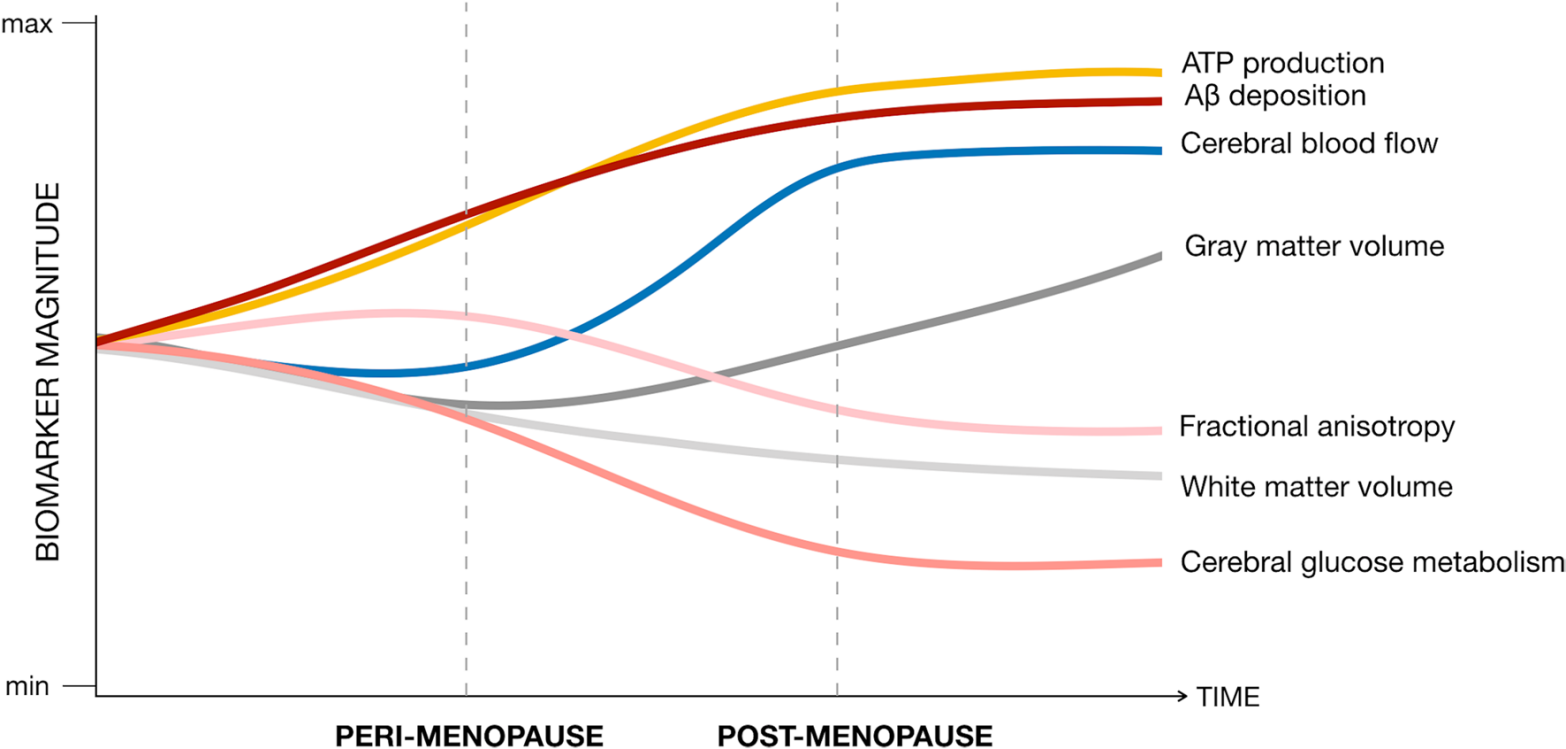
Summary of brain biomarker effects during the menopause transition. This figure summarizes the main results of the study by mapping estimated brain biomarker outcomes from pre-menopausal to peri-menopausal and post-menopausal stages. Biomarker measures extracted from representative clusters for each modality are displayed on a standardized scale and normalized to pre-menopausal levels to enable examination of the magnitude of biomarker effects by menopausal stage and across modalities.

The main biomarker findings of this study are summarized in Fig. 5.

### Sensitivity analysis

We performed a sensitivity analysis to test for effects of HT use and hysterectomy status on biomarker data. Results are presented in Supplementary Appendix. Briefly, including HT use and hysterectomy status as covariates left MT effects on brain biomarkers substantially unchanged. As compared to HT users, non-users exhibited lower GMV in inferior temporal gyrus, and lower CMRglc in parietal regions, independent of hysterectomy status. Restricting analysis to HT non-users confirmed results from the entire cohort, with two exceptions: CBF differences between MT groups were more pronounced among non-users, and FA differences between PERI and POST groups did not reach significance.

### Associations between biomarkers and cognition

There were no significant differences in cognitive scores between MT groups (Table 1), or between each MT group and the corresponding age-matched male group (Supplementary Table S12). As shown in Supplementary Table S13, in the POST group, precuneus GMV was positively associated with memory scores at cross-section (r = 0.311, *p* = 0.007) and longitudinally (r = 0.521, *p* = 0.032). ATP/PCr in temporo-parietal regions was positively associated with global cognition (Rho = 0.323, *p* = 0.027). There were no significant associations between cognitive scores and regional biomarkers among PRE and PERI groups (Supplementary Table S13).

## Discussion

Outcomes of this multi-modality neuroimaging analyses demonstrate that MT stage has pronounced effects on human brain’s structure, connectivity, and energy metabolism, and provide a neurological framework for both vulnerability and resilience. Effects were most pronounced in regions subserving higher-order cognitive processes, and were independent of age, APOE-4 status, HT usage, and hysterectomy status. Comparisons to age-matched males provided further evidence that neuroimaging biomarker effects were specific to menopausal endocrine aging rather than to chronological aging. In most brain regions and across modalities, brain biomarkers stabilized or recovered post-menopause. Cognitive preservation post-menopause correlated with GMV recovery and brain ATP production, highlighting possible compensatory mechanisms. In parallel to the adaptive process, Aβ deposition was more pronounced in PERI and POST women positive for APOE-4 genotype, indicating specificity for AD risk with onset in the peri-menopause.

The MT is a normal physiological event. However, while the majority of women undergo menopause without long-term adverse effects^1^, many are vulnerable to the neurological shifts that can occur during this transition, experiencing bothersome symptoms^2^ as well as a higher risk of depression, anxiety, and AD^13^. Preclinical work indicates that the brain has the ability to compensate for changes in estrogen levels and estrogen receptor activity during the MT^1^. In some instances, however, compensatory responses are diminished, lacking, or limited to some estrogen-regulated networks^1^, which may account for the complex MT phenotype observed in women^2^. Investigation of compensatory responses is still in the early stages^1^, though clinical observations suggest that gradual hormonal changes during spontaneous MT may allow “brain resetting”, e.g. neuronal adaptations to the hypo-estrogenic post-menopausal state^1,14,15^. Brain adaptation may account for the easing of menopausal symptoms like hot flashes, which tend to resolve 2–7 years into menopause^2^. Present neuroimaging results provide novel neurophysiological evidence for post-menopausal brain adaptation in humans, encompassing brain structure, connectivity and bioenergetics, and preservation of cognitive function.

Our results have several implications. First, compared to age-matched males, POST and PERI groups exhibited lower GMV in several cortical regions and subcortical structures such as hippocampus, amygdala, and thalamus. However, GMV generally stabilized post-menopause and selectively recovered in precuneus, an associative cortical area involved in social processes, episodic memory, and information integration^16^. On average, in the POST group, GMV in precuneus (a) was higher than the PERI group, (b) was comparable to age-matched males, and (c) increased over a 2-year span, a change that was not observed in males, indicating POST-specific recovery. Further, among POST participants, GMV in precuneus correlated with memory scores at cross-section and longitudinally, indicating that this area undergoes structural changes that are influenced by MT and have cognitive implications. Brain imaging studies of pregnancy, the other female-specific neuro-endocrine transition, also report dynamic GMV changes in precuneus, which are thought to reflect reduced neurogenesis in late pregnancy, followed by restoration by the time of weaning^17^. Although neurogenesis decreases with aging^18^, similar mechanisms may play a role in the brain’s adaptation to the post-menopausal stage. Notably, the observed MT-related anatomical pattern maps onto estrogen-regulated systems^1^ and displays notable similarities to the neural networks impacted by pregnancy^19^.

MT stage also influenced WMV within major tracts connecting extensive parts of the cerebral cortex and subcortical regions. Both POST and PERI groups exhibited widespread WMV loss compared to age-matched males, with the POST group also exhibiting lower WMV in corona radiata compared to PRE and PERI groups. In spite of the WMV loss, all MT groups exhibited higher FA than males in corona radiata, as well as in fornix of the POST group, and in fornix, uncinate fasciculus and external capsule of the PERI group. PERI and PRE groups also exhibited some clusters with lower FA than males, involving chiefly SLF, whereas the POST group showed no deficits. To our knowledge, there are no previous DTI studies of the MT. However, there is evidence for sex differences in WM microstructure that vary with age and endocrine aging status. Most DTI studies on the entire age range found that males have generally higher FA than females in WM tracts such as SLF^20^, as in our comparisons with PERI and PRE groups. In contrast, during puberty (the first neuro-endocrine transition for both genders), females display higher FA than males in several WM tracts including corona radiata and fornix^21^. Animal studies report that pregnancy is also accompanied by increased regional water diffusivity^22^. While FA has a number of determinants, the principle factors are myelination and tissue architecture, with the inference that higher FA represents more “efficient” WM organization^12^. As such, our findings suggest greater efficiency of a smaller corona radiata and fornix post-menopause, raising the possibility that MT is accompanied by further refinement of these regions’ connectivity. Longitudinal studies are warranted to map FA changes during the MT and in relation to other biomarkers.

The MT also impacted brain energetics on multiple levels. The POST group, and to a lesser extent the PERI group, exhibited hypometabolism in parieto-temporal cortices, consistent with previous reports in women at risk for AD^9–11^. However, in the present study, regional CMRglc largely plateaued post-menopause, suggesting adaptation to a new metabolic baseline after prolonged estrogen deficiency. Additionally, CBF and ATP production in temporo-parietal regions were elevated post-menopause, and ATP levels positively correlated with global cognition. Our findings of higher CBF in women than in men are consistent with the literature^23^, and further indicate that MT modulates CBF sex differences in midlife. To our knowledge, there are no previous studies of MT effects on brain ATP production in humans. Altogether, present results provide novel evidence for in vivo neurovascular-neurometabolic dissociations during the MT. Typically, regional brain activity, CMRglc, and CBF are coupled^24^. Dissociation can occur with aging, pathology, and inflammation^24^, or as a compensatory response^25^. While data in humans are scarce, preclinical evidence indicates that estrogen loss during MT triggers CMRglc declines^26^, prompting an adaptive reaction to increase ketone bodies utilization as an alternative fuel for ATP^27^. Continued reliance on ketones leads to compromised mitochondrial function, WM catabolism, and cellular apoptosis in animals^26,27^. In light of preclinical work, the higher CBF and ATP levels observed in our POST women may reflect a compensatory reaction to glucose hypometabolism, as well as a means to increase ketone metabolism.

We previously reported lower peripheral mitochondrial cytochrome oxidase activity (COX) in POST versus PRE women at risk for AD^28^. Herein, we investigated mitochondria ATP production in *brain*, which was higher in POST women independent of APOE-4 status, suggesting different mitochondrial activity profiles in brain and periphery. Animal models of menopause show dynamic relationships between central and peripheral metabolic systems, which tend to shift from uncoupled to coupled under metabolic stress^29^. Although brain mitochondria might eventually falter in older POST women, the positive associations between ATP production and global cognition suggest a recovery mechanism, at least in midlife. As aging and menopause differ to some extent between humans and animals, the neuro-energetics of menopause are also likely to differ – in this case, by supporting women’s ability to transition into late life with preserved cognition, as discussed below.

In parallel to the hypothesized adaptive process, POST and PERI groups, especially APOE-4 carriers, exhibited higher Aβ deposition compared to the PRE group and to age-matched males. While Aβ deposition was mild, this data is consistent with previous studies of women at risk for AD^9–11^, and support evidence that interactions between age, female gender and APOE-4 increase AD vulnerability during peri-menopause^30^. Chronologically, MT maps onto the preclinical phase of AD, which begins decades prior to symptom onset^31^. The earlier onset of, and longer exposure to Aβ pathology may help explain the higher prevalence of AD in females, with POST women comprising over 60% of all cases^32^. It warrants emphasis that reproductive aging is not uniformly associated with AD risk. While some women in our cohort might eventually develop AD, for others, Aβ deposition could reflect accelerated biological aging due to hormonal declines instead^33^. In fact, over 20% of healthy elderly display moderate cerebral Aβ burden and no dementia^31^.

The proportion of APOE-4 carriers in our study was 42%, which is higher than the 15–30% observed in the general population^34^. Many of our participants volunteer because of concerns about cognitive functioning, which tend to be more frequent among APOE-4 carriers^35^. As such, our cohort may be self-enriched with individuals at higher a priori risk of AD. Although our results were independent of APOE-4 status, more studies are needed to replicate these findings in community-dwelling individuals randomly recruited from the population.

The observed MT effects were independent of HT and hysterectomy status, though HT use was associated with mild beneficial effects on GMV and CMRglc, consistent with some clinical trials^6^. Whether HT provides protection against cognitive aging and AD remains unclear. Observational studies generally found positive effects on cognition across multiple HT regimens^36^, whereas clinical trials of late POST women aged 65 or older reported an increased dementia risk with estrogen-plus-progestin HT, and no effects with estrogen-alone^37^, while studies of early POST women observed no adverse or beneficial effects on cognition^38,39^. Overall, HT’s efficacy is thought to depend on timing of treatment initiation with respect to age at menopause, with benefits pertaining to early initiation, especially after induced menopause^7,37^. Our neuroimaging results point to the MT as a dynamic neurological process and, therefore, a window of vulnerabilities and opportunities when the human brain is influenced by biochemical adjustments, but is also likely to be receptive to interventions. More studies are needed to test the efficacy of HT instituted prior to menopause, and in relation to brain biomarker levels.

In our study, POST participants did not exhibit impaired cognitive performance as compared to the other groups. While self-reports of poor memory and concentration are common in women undergoing MT^2^, menopause itself hasn’t been associated with clinically significant functional impairment or deficits on cognitive testing^40^. Further, it is well-documented that women perform better than men on several cognitive domains across the adult lifespan– an advantage that seems to persist even after a dementia diagnosis^41^. Lack of cognitive decline may seem in contrast with neuroimaging findings of extensive MT-stage effects on brain biomarkers. In the present study, GMV recovery and ATP production positively correlated with cognitive scores in the POST group. While this awaits confirmation, present results provide neurophysiological insights on brain adaptation during the MT, which may at least in part, account for the lack of cognitive impairment among POST women^40^. Large-scale epidemiological studies reported a decrement in cognitive performance during the peri-menopause, followed by a rebound to pre-menopausal levels in post-menopause^42^. This is consistent with the post-menopausal brain biomarker recovery observed in our study. Additionally, studies that differentiated between early and late menopausal stages reported subtle yet transitory cognitive changes during the MT^43^, which have yet to be investigated with neuroimaging. However, it is possible that brain and cognitive aging trajectories may differ among MT women depending on genetic predisposition, medical history, and environmental influences. More longitudinal imaging studies with larger samples are warranted to address these questions. Additionally, as our cohort was highly educated, the observed lack of cognitive deficits may not be generalizable to women with different educational or socio-economical background.

Another limitation of this study is the low percentage of minority participants, which limits the generalizability of our findings. Today, no brain imaging studies have been conducted to investigate MT effects on brain biomarkers based on ethnicity. Multiple studies have indicated increased frequency and severity of menopausal symptoms, particularly vasomotor symptoms, with greater prevalence in African-American and Hispanic women^44^, which strongly argues for studies that specifically address differences in MT-associated biomarker outcomes across ethnic groups.

From a methodological perspective, we examined statistically powered groups of women at different MT stages, paired with age correction procedures including comparisons to age-matched males and longitudinal evaluations in a subset of participants. Nonetheless, a causal link between MT and brain biomarkers cannot be unequivocally established. We chose this study design because the timing of menopause is highly variable, with a median age at menopause of 51 years, and a distribution of 40–58 years^2^. Longitudinal studies may require > 10 years of follow-ups to capture the effects of MT on brain biomarkers. While studies of surgical menopause ideally reduce follow-up times, the procedure seems associated with different, possibly more severe outcomes^45,46^. Longitudinal studies are warranted to replicate our findings and test for differential effects of induced and spontaneous menopause.

Determination of MT status was based on established diagnostic criteria^47^. Nonetheless, some PERI may have been early POST, while some early POST could have been late PERI. This would, however, conservatively reduce power in detecting MT-stage effects. Considering the hormonal changes women experience during MT, and the observed biomarker effects, we attribute our results to the endocrine climate of menopause. Our analyses were corroborated by testing of males of similar demographic and socio-economical characteristics. Future studies tracking changes in hormones, medical status, and lifestyle are needed to clarify the complex relationships between MT and brain aging^13^, and identify which factors impact successful versus unsuccessful brain adaptation post-menopause.

Overall, present findings show that human menopause is a dynamic neurological transition that reshapes the neural landscape of the female brain during midlife endocrine aging, and provide preliminary evidence for an adaptive process serving the transition into late life.

## Methods

### Participants

This is a non-randomized natural history non-treatment study of healthy, cognitively normal female and male participants ages 40–65 years at different menopausal stages, recruited at Weill Cornell Medicine (WMC) and NYU School of Medicine between 2015 and 2020. Participants were recruited by self-referral, flyers, and word of mouth, as described^9–11^. Pre-established exclusion criteria included medical conditions that may affect brain structure or function (e.g. stroke, any neurodegenerative diseases, major psychiatric disorders, hydrocephalus, white matter lesions suggestive of demyelinating disease such as Multiple Sclerosis, intracranial mass and infarcts on MRI), use of psychoactive medications, and contraindications to MRI or PET. Participants had Mini Mental Status Exam ≥ 27 and normal cognitive performance for age and education^9–11^.

The patients’ sex was determined by self-report. APOE genotype was assessed using quantitative Polymerase Chain Reaction (qPCR) procedures^9–11^. Participants carrying one or two copies of APOE-4 allele were grouped as carriers, and compared to non-carriers.

#### Standard protocol approvals, registrations, and patient consents

All methods were carried out in accordance with relevant guidelines and regulations. All experimental protocols were approved by the WMC and NYU Institutional Review Boards. Written informed consent was obtained from all participants.

#### Cognitive testing

Our neuropsychological testing battery included tests measuring three cognitive domains: memory (immediate and delayed recall of a paragraph and paired associates), higher-order processing (block design tests), and language (object naming)^9–11^. We computed (i) a global cognitive score by creating Z-scores within each domain and averaging across the domains; and (ii) a memory score by creating Z-scores for each memory test and averaging across tests.

#### Menopause assessments

Determination of menopausal status was based on the Stages of Reproductive Aging Workshop (STRAW) criteria^47^ and corroborated by means of hormone assessments. Female participants were classified as regular cyclers (PRE), irregular cyclers (PERI), and no cycle for 12 or more months (POST).

### Brain imaging acquisition and analysis

All participants underwent structural MRI, and 160 participants received ^18^F-FDG and ^11^C-PiB PET at WMC. In 2017, we started acquiring DTI, ASL, and MRS scans, which have been done on 100 participants.

Our protocol included three MRI sequences on a 3.0 T G.E. Discovery MR750 scanner: (a) sagittal T1-MRI [Brain Volume Imaging (BRAVO); 1.0 × 1.0 ×1.0 mm resolution, 8.2 ms repetition time (TR), 3.2 ms echo time (TE), 25.6 cm field of view (FOV), 256 × 256 matrix] used to estimate GMV and WMV; (b) DTI scan [b = 0 s/mm^2^, b = 1000 s/mm2 55-directions, 8000 ms TR , 65 ms TE, 256 × 256 matrix, 0.9 × 0.9 ×1.8 mm resolution]; (c) ASL scan [pseudo-continuous technique with 4851 ms TR, 10.6 ms TE, 4 averages, 24 cm FOV, 2.0 × 2.0 × 3.8 mm resolution] used to estimate CBF using arterial blood water^48^.

^31^P-MRS was acquired on the GE scanner using a dual tuned 32-channel ^31^P/^1^H quadrature head coil [2048 points, 5000 Hz sweep width, 2000 ms TR, 2 averages, 55° flip angle at 51.3 MHz, 24 cm FOV] to assess mitochondrial function in brain through the mapping of intracellular ATP and PCr levels^49,50^. A higher ATP/PCr ratio reflects greater ATP production relative to utilization^51^. Shimming was performed using a ^1^H single voxel technique placed over the entire brain. Raw data was processed using Hamming and Fermi k-space filters, 20 Hz exponential filtering and zero-filling in time, x and y-domains prior to 3D Fast Fourier Transformation. The PCr peak is set at 0.0 ppm and susceptibility corrections performed. Baseline correction was applied by an experienced analyst (JPD). This resulted in a 16 × 16 image of 1.5 × 1.5 × 3.0 cm voxels with the signal intensity in each voxel corresponding to the peak area of the ^31^P metabolite. The central 4 slices of Chemical Shift Imaging (CSI) data were then registered to the BRAVO sequence.

PET scans were acquired on a Siemens BioGraph mCT 64-slice PET/CT operating in 3D mode [70 cm transverse FOV, 16.2 cm axial FOV] following standardized procedures^9–11^. Summed images were obtained 40–60 min post-injection of 5 mCi of ^18^F-FDG, and 60–90 min post-injection of 15 mCi of ^11^C-PiB. All images were corrected for attenuation, scatter and decay, and smoothed for uniform resolution^52^.

Two-year follow-up volumetric MRI and FDG-PET scans were performed on 17 POST women (age at baseline scan: 57 ± 3 years; time to follow-up: 2.2 ± 0.4 years) and 12 males within the same age range (age at baseline: 56 ± 4 years, time to follow-up: 2.4 ± 0.4 years) using the same protocol and equipment as the baseline exams.

#### Multiparametric mapping

All images were processed in Statistical Parametric Mapping (SPM12, (https://www.fil.ion.ucl.ac.uk/spm/software/spm12/) and Matlab 7.8, using a fully automated image processing pipeline^9–11^. For each participant, scans were co-registered to the T1-MRI and to each other using the Normalized Mutual Information routine^53^. Volumetric scans were processed with voxel-based morphometry (VBM), including Jacobian modulation to restore volumes using the unified segmentation algorithm, DARTEL normalization of the segments, and application of an 8 mm full-width at half maximum (FWHM) smoothing kernel^53^. Co-registered DTI, ASL, and PET scans were spatially normalized using subject-specific transformation matrices obtained from the corresponding MRI and smoothed at 10-mm FWHM. For longitudinal analysis, each participant’s MRIs were processed using longitudinal routines incorporating rigid-body registration, intensity inhomogeneity correction, and nonlinear diffeomorphic registration^53^. Co-registered follow-up PET were processed using the baseline MRI as the anchor.

ATP/PCr and PiB uptake were quantified using FreeSurfer 6.0 and Desikan-Killiany Atlas-based cortical ROIs^54,55^ applied to the aligned MRI. For PiB analysis, we created an AD-mask by averaging parietal, temporal, frontal, posterior cingulate and precuneus ROIs^56,57^. PiB uptake in AD-mask was normalized to cerebellar GM uptake obtained via FreeSurfer.

### Statistical analysis

Analyses were performed in SPSS v.25 and SPM12. Clinical, demographic and cognitive measures were examined with General Linear Models (GLM) or Chi-squared tests at *p* < 0.05.

To address the age difference between POST and PRE groups we^11,58^: (a) used box plots and frequency diagrams to ensure that we had sufficient age overlap among women of different MT statuses, which enabled us to examine the effects of endocrine aging separately from those of chronological aging; (b) included age as a covariate in all analyses; (c) compared each MT group to an age-range matched male group (MALE_PRE,_ MALE_PERI,_ MALE_POST_); and (d) compared 2-year MRI and FDG-PET changes in a subset of POST women and MALE_POST_.

All images were analyzed using SPM12, except for MRS and PiB-PET ROI data which were examined using SPSS, as described below. SPM12 analyses were adjusted by age, APOE-4 status, and modality-specific confounds, e.g. GMV, WMV and FA were adjusted by total intracranial volume (TIV) obtained via Freesurfer, CMRglc by global metabolic activity, CBF by global CBF. Statistical maps were conservatively obtained by first applying an a priori masking image including regions involved in the brain estrogen network with known cognitive functions^1^, and then a stringent cluster-level FWE correction at *p* < 0.05, with cluster extent ≥ 20 voxels. The masking image comprised anterior and posterior cingulate cortex; fusiform gyrus; inferior and medial orbitofrontal cortex; inferior, medial, middle and superior frontal cortex; inferior, middle, and superior temporal gyrus; inferior and superior parietal lobule; insula; medial temporal lobe; precuneus; putamen; and thalamus. For WM analysis, the brainstem was also included. Further, results were examined after application of an a priori GM or WM mask to restrict analysis to GM or WM fiber voxels, respectively. Identification of GM clusters was made using MNI coordinates, and of WM clusters by reference to the Johns Hopkins University White-Matter Labels atlas^59^.

In figures, statistical parametric maps and surface maps were obtained using SPM12 and SurfRend v.1 (http://spmsurfrend.sourceforge.net/).

#### Biomarker differences between MT groups

For SPM12 analyses, we used full factorial models to test for biomarker differences between MT groups. If a significant main effect was found, we then proceeded to separately examine the directionality of biomarker differences between paired groups using post-hoc *t*-contrasts. For all biomarker modalities, statistical parametric maps of significant results were saved as masking images, which were then used as implicit masks of MT stage-specific regional biomarker effects in subsequent analyses (see b and c, below). For example, the POST group showed larger GMV in precuneus compared to PERI. The precuneus cluster was saved as a masking image for further examination of POST and PERI-specific GMV effects in this region.

For ROI analyses, we used (i) mGLMs with post-hoc Sidak tests to test for group differences in ATP/PCr in regions showing MT effects on CMRglc and CBF (e.g. temporo-parietal regions), adjusting by age and APOE-4 status as covariates, at *p* < 0.05; and (ii) GLMs with post-hoc Sidak tests to examine AD-mask PiB uptake for MT group effects, and for APOE-4 effects by (i) examining APOE-4 status as a covariate; (ii) testing for interactions between MT and APOE-4 status; and (iii) for MT effects within each APOE-4 subgroup, at *p* < 0.05.

#### Comparisons to age-matched males

For SPM12 analyses, we used two-sample t-tests to compare each MT group to the corresponding age-matched male group, using the same confounders as above. For all biomarker modalities, our main endpoint was detection of sex-related biomarker differences within the implicit masks of MT stage-specific effects described in (a). For example, we used the precuneus cluster described above to test for GMV differences between POST and PERI groups and corresponding age-matched male groups. Comparison to MALE_PERI_ confirmed presence of lower precuneus GMV in the PERI group, while comparison to MALE_POST_ showed no significant differences in precuneus of the POST group, suggesting POST-specific GMV recovery. As this analysis was restricted to specific clusters, our secondary endpoint was detection of group differences in the entire search volume.

For ROI analyses, we used (i) mGLMs with post-hoc Sidak tests to test for ATP/PCr group differences in regions showing CMRglc and CBF differences between POST and MALE_POST_; and (ii) GLMs with post-hoc Sidak to examine AD-mask PiB uptake for group effects and for APOE-4 status effects (as described above), at *p* < 0.05.

#### Biomarker changes post-menopause

We investigated whether the structural and CMRglc effects of menopause were persistent 2-years after the first examination in a subset of POST and MALE_POST_. Voxel-based changes in GMV, WMV and CMRglc were examined by means of post-hoc *t*-tests on the baseline versus 2-year follow-up scans for each group, and by comparing the maps of change between groups, using SPM12. This analysis allowed us to identify brain regions showing (i) significant biomarker changes over time in each group, and (ii) different rates of change in POST versus MALE_POST_.

Our main endpoint was detection of biomarker changes within the implicit masks of POST-specific regional effects, as described in (a). Therefore, we first tested for longitudinal changes within those pre-specified regions of the POST group, and then compared the rate of change in those regions between POST and MALE_POST_. For example, the POST group showed larger precuneus GMV compared to PERI. Longitudinally, the POST group showed increased precuneus GMV over time, whereas MALE_POST_ did not, supporting evidence of POST-specific GMV recovery in this region. We then tested for longitudinal differences in the entire search volume, e.g. biomarker changes outside of the implicit masks. Results were examined at *p* < 0.05, cluster-level corrected, adjusting for time to follow-up and modality-specific confounds.

#### Cognitive measures: group effects and biomarker associations

We examined cognitive measures for differences across groups using Kruskal–Wallis non-parametric tests for global cognition as this measure did not follow a normal distribution, and GLMs with post-hoc Sidak tests for analysis of memory scores, adjusting by age, education, and APOE-4 status, at *p* < 0.05. Cognitive measures were then tested for correlations with biomarker data. Since cognitive measures were preserved in the POST group, we tested for associations between cognitive measures and regional biomarkers showing preservation in the POST group, e.g. precuneus GMV, temporo-parietal ATP/PCr and CBF. For descriptive purposes, we examined these associations also among PRE and PERI groups. Spearman’s Rho tests were used to test for correlations with global cognition, and Pearson’s r tests for correlations with memory scores, at *p* < 0.05, 2-tailed.

## Supplementary Information

Supplementary Information 1.

Supplementary Information 2.

## Data Availability

The datasets analyzed during the current study may be made available from the corresponding author on reasonable request.

## References

[1] Brinton RD, Yao J, Yin F, Mack WJ, Cadenas E (2015). Perimenopause as a neurological transition state. Nat Rev Endocrinol.

[2] Monteleone P, Mascagni G, Giannini A, Genazzani AR, Simoncini T (2018). Symptoms of menopause—global prevalence, physiology and implications. Nat Rev Endocrinol..

[3] McEwen BS, Alves SE, Bulloch K, Weiland NG (1997). Ovarian steroids and the brain: implications for cognition and aging. Neurology.

[4] Yue X (2005). Brain estrogen deficiency accelerates Abeta plaque formation in an Alzheimer's disease animal model. Proc Natl Acad Sci U S A.

[5] Yao J (2009). Mitochondrial bioenergetic deficit precedes Alzheimer's pathology in female mouse model of Alzheimer's disease. Proc Natl Acad Sci U S A.

[6] Comasco E, Frokjaer VG, Sundström-Poromaa I (2014). Functional and molecular neuroimaging of menopause and hormone replacement therapy. Front Neurosci.

[7] Maki PM (2008). The timing of estrogen therapy after ovariectomy—implications for neurocognitive function. Nat Clin Pract Endocrinol Metabol.

[8] Bertram L, Tanzi RE (2008). Thirty years of Alzheimer's disease genetics: the implications of systematic meta-analyses. Nat Rev Neurosci.

[9] Mosconi L, Rahman A, Diaz I, Wu X, Scheyer O, Hristov H, Vallabhajosula S, Isaacson R, Leon M, Brinton R (2018). Increased Alzheimer's risk during the menopause transition: a 3-year longitudinal study. PloS One.

[10] Mosconi L (2017). Sex differences in Alzheimer risk Brain imaging of endocrine vs chronologic aging. Neurology.

[11] Rahman A (2020). Sex-driven modifiers of Alzheimer risk. Neurology.

[12] Le Bihan D (2001). Diffusion tensor imaging: concepts and applications. J Magn Reson Imaging.

[13] Rahman A (2019). Sex and gender driven modifiers of Alzheimer’s: the role for estrogenic control across age, race, medical, and lifestyle risks. Front Aging Neurosci.

[14] Deecher DC, Dorries K (2007). Understanding the pathophysiology of vasomotor symptoms (hot flushes and night sweats) that occur in perimenopause, menopause, and postmenopause life stages. Arch Womens Ment Health.

[15] Rossmanith WG, Ruebberdt W (2009). What causes hot flushes? The neuroendocrine origin of vasomotor symptoms in the menopause. Gynecol Endocrinol.

[16] Cavanna AE, Trimble MR (2006). The precuneus: a review of its functional anatomy and behavioural correlates. Brain.

[17] Hoekzema E (2017). Pregnancy leads to long-lasting changes in human brain structure. Nat Neurosci..

[18] Gross CG (2000). Neurogenesis in the adult brain: death of a dogma. Nat Rev Neurosci..

[19] Brunton PJ, Russell JA (2008). The expectant brain: adapting for motherhood. Nat Rev Neurosci.

[20] Kanaan RA (2012). Gender differences in white matter microstructure. PLoS ONE.

[21] Bava S (2011). Sex differences in adolescent white matter architecture. Brain Res.

[22] Chan RW (2015). Structural and functional brain remodeling during pregnancy with diffusion tensor MRI and resting-state functional MRI. PLoS ONE.

[23] Aanerud J (2012). Brain energy metabolism and blood flow differences in healthy aging. J Cerebr Blood Flow Metabol.

[24] Iadecola C (2004). Neurovascular regulation in the normal brain and in Alzheimer's disease. Nat Rev Neurosci.

[25] Masamoto K (2016). New horizons in neurovascular coupling: a bridge between brain circulation and neural plasticity, 1st edn.

[26] Brinton RD (2008). The healthy cell bias of estrogen action: mitochondrial bioenergetics and neurological implications. Trends Neurosci.

[27] Ding F, Yao J, Rettberg JR, Chen S, Brinton RD (2013). Early decline in glucose transport and metabolism precedes shift to ketogenic system in female aging and Alzheimer's mouse brain: implication for bioenergetic intervention. PLoS ONE.

[28] Mosconi L (2017). Perimenopause and emergence of an Alzheimer’s bioenergetic phenotype in brain and periphery. PloS One.

[29] Wang Y (2020). Midlife chronological and endocrinological transitions in brain metabolism: system biology basis for increased Alzheimer’s risk in female brain. Sci Rep.

[30] Riedel BC, Thompson PM, Brinton RD (2016). Age, APOE and sex: triad of risk of Alzheimer's disease. J Steroid Biochem Mol Biol.

[31] Sperling RA, Karlawish J, Johnson KA (2013). Preclinical Alzheimer disease-the challenges ahead. Nat Rev Neurol..

[32] Ferretti MT (2018). Sex differences in Alzheimer disease—the gateway to precision medicine. Nat Rev Neurol.

[33] Levine ME (2016). Menopause accelerates biological aging. Proc Natl Acad Sci.

[34] Singh PP, Singh M, Mastana SS (2006). APOE distribution in world populations with new data from India and the UK. Ann Hum Biol..

[35] Small GW (1999). Memory self-appraisal in middle-aged and older adults with the apolipoprotein E-4 allele. Am J Psychiatry.

[36] Morrison JH, Brinton RD, Schmidt PJ, Gore AC (2006). Estrogen, menopause, and the aging brain: how basic neuroscience can inform hormone therapy in women. J Neurosci..

[37] Lobo RA (2017). Hormone-replacement therapy: current thinking. Nat Rev Endocrinol..

[38] Miller VM (2019). The Kronos early estrogen prevention study (KEEPS): what have we learned?. Menopause.

[39] Henderson VW (2016). Cognitive effects of estradiol after menopause: a randomized trial of the timing hypothesis. Neurology.

[40] Maki PM, Henderson VW (2016). Cognition and the menopause transition. Menopause.

[41] Rentz DM (2017). Sex differences in episodic memory in early midlife: impact of reproductive aging. Menopause.

[42] Greendale GA (2009). Effects of the menopause transition and hormone use on cognitive performance in midlife women. Neurology.

[43] Weber MT, Rubin LH, Maki PM (2013). Cognition in perimenopause: the effect of transition stage. Menopause.

[44] Obermeyer CM (2000). Menopause across cultures: a review of the evidence. Menopause.

[45] Rocca WA, Grossardt BR, Shuster LT (2014). Oophorectomy, estrogen, and dementia: a 2014 update. Mol Cell Endocrinol..

[46] Zeydan B (2019). Association of bilateral salpingo-oophorectomy before menopause onset with medial temporal lobe neurodegeneration. JAMA Neurol..

[47] Harlow SD (2012). Executive summary of the stages of reproductive aging workshop + 10: addressing the unfinished agenda of staging reproductive aging. Menopause.

[48] Wang J (2003). Arterial transit time imaging with flow encoding arterial spin tagging (FEAST). Magn Reson Med.

[49] Rijpma A, van der Graaf M, Meulenbroek O, Olde Rikkert MGM, Heerschap A (2018). Altered brain high-energy phosphate metabolism in mild Alzheimer's disease: a 3-dimensional 31P MR spectroscopic imaging study. NeuroImage Clin.

[50] Du F (2008). Tightly coupled brain activity and cerebral ATP metabolic rate. Proc Natl Acad Sci..

[51] Pettegrew JW (1988). Correlation of phosphorus-31 magnetic resonance spectroscopy and morphologic findings in Alzheimer's disease. Arch Neurol.

[52] Joshi A, Koeppe RA, Fessler JA (2009). Reducing between scanner differences in multi-center PET studies. Neuroimage.

[53] Ashburner J, Friston KJ (2000). Voxel-based morphometry—the methods. Neuroimage.

[54] Desikan RS (2006). An automated labeling system for subdividing the human cerebral cortex on MRI scans into gyral based regions of interest. Neuroimage.

[55] Fischl B (2012). FreeSurfer. NeuroImage.

[56] Vlassenko AG (2016). Imaging and cerebrospinal fluid biomarkers in early preclinical alzheimer disease. Ann Neurol..

[57] Mintun MA (2006). [11C]PIB in a nondemented population: potential antecedent marker of Alzheimer disease. Neurology.

[58] Becker JB (2005). Strategies and methods for research on sex differences in brain and behavior. Endocrinology.

[59] Hua K (2008). Tract probability maps in stereotaxic spaces: analyses of white matter anatomy and tract-specific quantification. Neuroimage.

