# Supplementary material for: Menopause impacts human brain structure, connectivity, energy metabolism, and amyloid-beta deposition
Source: Sci Rep. 2021 Jun 9;11:10867. doi: 10.1038/s41598-021-90084-y (PMC8190071; doi:10.1038/s41598-021-90084-y)
Supplement: Supplementary file 1 — Supplementary Information 1. [file 41598_2021_90084_MOESM1_ESM.docx]

# Menopause impacts human brain structure, connectivity,

# energy metabolism, and amyloid-beta deposition

Lisa Mosconi, PhD^1,2,3*^, Valentina Berti, MD, PhD^4^, Jonathan Dyke, PhD^2^, Eva Schelbaum, MA^1^, Steven Jett, BA^1^, Lacey Loughlin, BA^1^, Grace Jang, BA^1^, Aneela Rahman, PhDc^1^, Hollie Hristov, NP^1^, Silky Pahlajani, MD^1,2^, Randolph Andrews, BS^5^, Dawn Matthews, PhD^5^, Orli Etingin, MD^6^, Christine Ganzer, PhD^7^, Mony de Leon, EdD^2^, Richard Isaacson, MD^1^, Roberta Diaz Brinton, PhD^8^.

# Supporting Information - Appendix

## METHODS

As shown in **Supplementary Table S1**, 32% of the post-menopausal (POST) women and 13% of the peri-menopausal (PERI) women reported taking menopause hormone therapy (HT) at the time of the evaluation (current users). Another 4% POST and 2% PERI women reported having taken HT in the past (past users). Nineteen percent of POST women and 6% PERI women reported having undergone a hysterectomy and/or oophorectomy. Herein, we performed a sensitivity analysis to examine the effects of HT status (users vs. non-users) and hysterectomy status (positive vs. negative history of hysterectomy/ oophorectomy) on brain biomarkers. We conducted three separate analyses:

1) We included HT status and hysterectomy status as confounders in all biomarker analyses comparing MT groups.

2) We divided the POST group into HT users (current and past) and non-users, and compared brain biomarkers between HT groups, adjusting for modality-specific confounds. For SPM analysis, results from general linear models with post-hoc t-tests were examined within the clusters affected by MT stage (e.g., using an implicit mask of the POST vs. PERI and PRE contrasts for each biomarker modality) at p<0.05 cluster-level small-volume corrected for Family-Wise Type Error (FWE). We used SPSS to examine ATP/PCr and PiB measures in the same ROIs as in the main analysis of MT groups, using multivariate and univariate general linear models, respectively, with post-hoc Sidek tests, at p<0.05. Analyses were repeated with adjustment for hysterectomy status. As only a minority (13%) of PERI women reported taking HT, we did not compare HT users and non-users in this group.

3) We repeated all biomarker comparisons of MT groups in the group of HT non-users.

## RESULTS

## Biomarker differences between MT groups adjusting for HT and hysterectomy status

### Structural measures

Including HT and hysterectomy status as covariates in the analyses of MT-stage effects on structural brain biomarkers, e.g. GMV, WMV, and FA, left results substantially unchanged (**Appendix** **Figure 1)**.

### **Appendix Figure 1**. Structural biomarker differences between MT groups adjusted by HT status


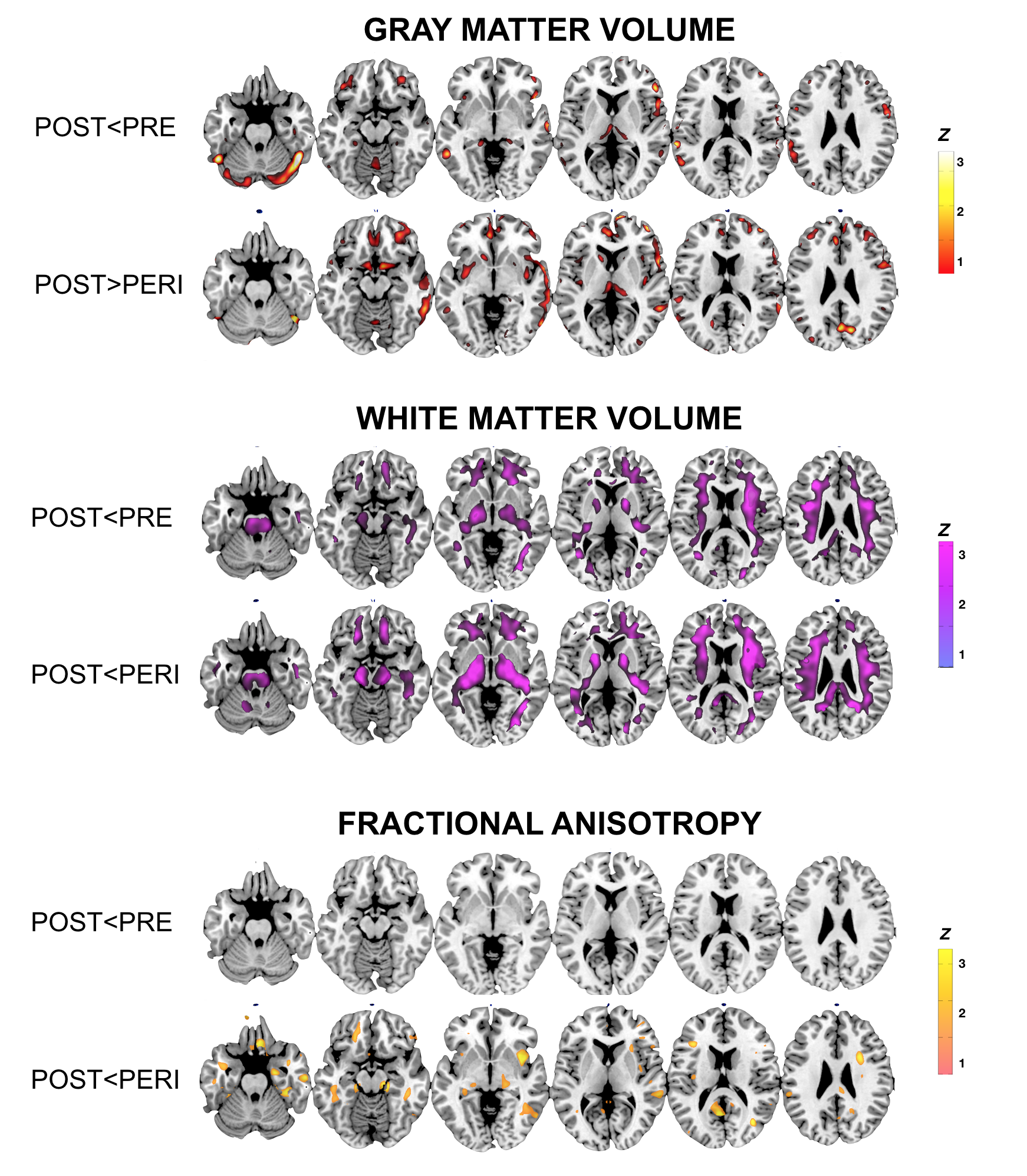


MRI slice overlays displaying, for each contrast:

1. GMV differences at a GM-matter masked threshold of p<0.05, cluster-level corrected for Family-Type Wise Error (FWE), adjusted for age and total intracranial volume (TIV).
2. WMV differences at a WM-matter masked threshold of p<0.05, cluster-level corrected for FWE, adjusted for age and TIV.
3. FA differences at a WM-matter masked threshold of p<0.05, cluster-level corrected for FWE, adjusted for age and TIV.

### Metabolic measures

Including HT and hysterectomy status as covariates in the analyses of MT-stage effects on CMRglc and CBF left results substantially unchanged (**Appendix Figure 2).**

#### **Appendix Figure 2**. FDG and CBF biomarker differences between MT groups adjusted by HT status


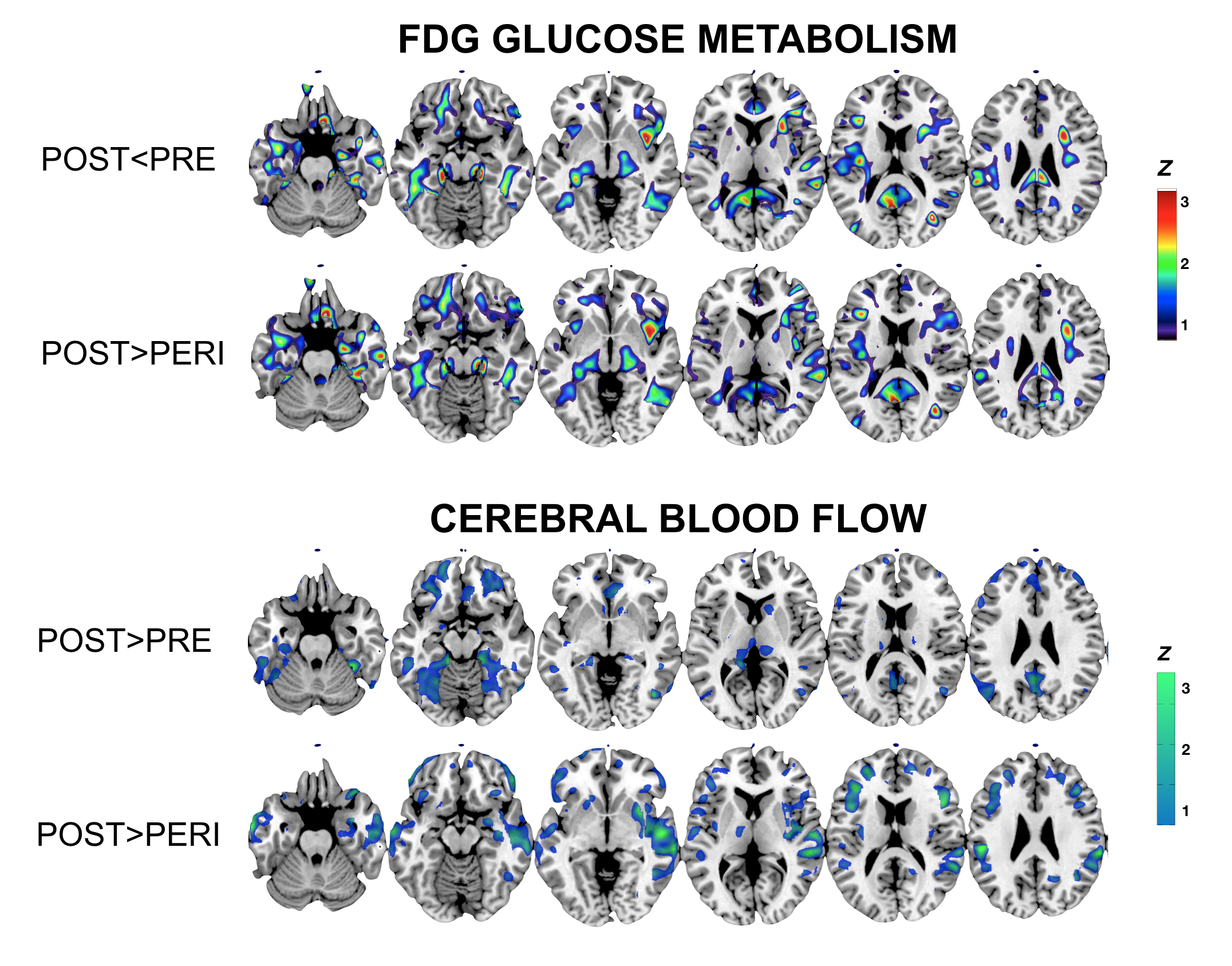


MRI slice overlays displaying, for each contrast:

1. Regional CMRglc differences at a GM-matter masked threshold of p<0.05, cluster-level corrected for FWE, adjusted for age and global CMRglc.
2. CBF differences at a GM-matter masked threshold of p<0.05, cluster-level corrected for FWE, adjusted for age and global CBF.

We used the brain regions showing MT-stage effects on CMRglc and CBF measures (e.g. superior, middle, and inferior temporal cortex, and inferior parietal lobule) to guide ROI selection for ATP/PCr measures. General linear models showed no significant effects of HT status and hysterectomy status on ATP/PCr measures, which remained overall higher in POST, intermediate in PERI, and lowest in PRE (**Appendix Table 1**).

#### **Appendix Table 1**. MRS ATP/PCr measures by MT group adjusted by age, HT status and hysterectomy status

|  | **PRE** | **PERI** | **POST** |
| --- | --- | --- | --- |
| Superior temporal gyrus | 1.15(0.04) | 1.18(0.03) | 1.24(0.02)* |
| Middle temporal gyrus | 1.11(0.03) | 1.14(0.02) | 1.19(0.02)* |
| Inferior temporal gyrus | 1.07(0.03) | 1.11(0.02) | 1.14(0.02) |
| Inferior parietal lobule | 0.76(0.05) | 0.76(0.03) | 0.83(0.03) |

Values are means (SE). *Different from the PRE group on univariate post-hoc analysis, p<0.05.

### Amyloid-β load

General linear models showed no significant effects of HT status and hysterectomy status on PiB uptake in AD-mask. Adjusting for age, HT status and hysterectomy status, the POST group had higher PiB uptake than PRE (p=0.005) and showed borderline effects compared to PERI (p=0.063; **Appendix Table 2**), as in the analysis with the full dataset.

#### **Appendix Table 2**. PiB-PET amyloid-β load by MT group adjusted by age, HT status and hysterectomy status

|  | **PRE** | **PERI** | **POST** |
| --- | --- | --- | --- |
| AD-mask SUVR | 0.99(0.14) | 1.09(0.07) | 1.31(0.08)* |

Values are means (SE), adjusted by age, HT status, and hysterectomy status. *Different from PRE group, p<0.05.Abbreviations: SUVR, standardized uptake value ratio to cerebellar gray matter PiB uptake.

## Longitudinal biomarker effects

Only 3 POST women with longitudinal scans reported taking HT. Descriptively, there was no clear evidence for HT users having more favorable biomarker rates than non-users (**Appendix Figure 3**). None of these women had undergone a hysterectomy.

#### **Appendix Figure 3**. Biomarker change by HT status


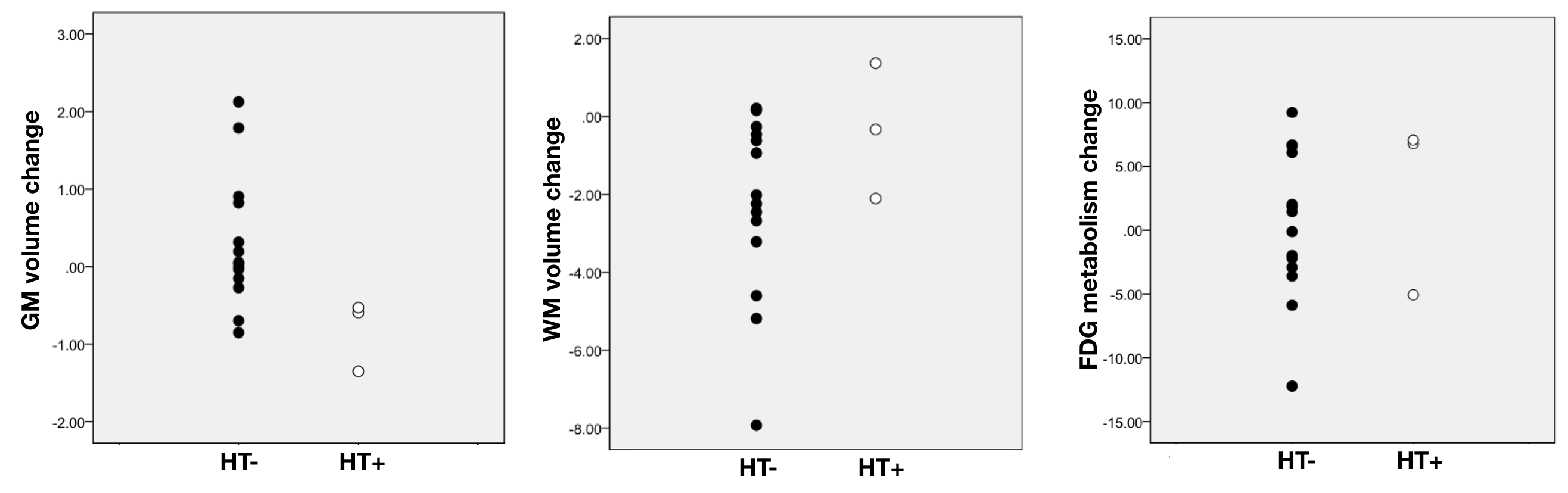


Abbreviations: HT, menopause hormonal therapy, users (HT+) vs non-users (HT-).

## Biomarker differences between HT users and non-users

The POST group included 47 HT non-users, 21 current users and 5 past users. Groups were comparable for demographical characteristics (**Appendix Table 3**). The frequency of hysterectomies was slightly higher in the HT-user group (25% vs. 15%), which did not reach statistical significance.

#### **Appendix Table 3**. Clinical characteristics by HT group

|  | **HT non-users** | **HT users** |
| --- | --- | --- |
| N | 42 | 29 |
| Baseline age, y | 58(4) | 56(4) |
| Education, y | 17(2) | 17(3) |
| Ethnicity, % White | 88 | 93 |
| *APOE* ε4 carrier status, % positive | 48 | 50 |
| Hysterectomy status, % positive | 15 | 25 |
| MMSE scores | 30(1) | 29(1) |

Values are means (SD), unless otherwise specified.

### Structural measures

Results are reported in **Appendix Table 4**. Adjusting for age and total intracranial volume, HT non-users showed lower GM volume in inferior temporal cortex as compared to HT users. There were no group differences for WM volume and FA measures. Results remained unchanged adjusting for hysterectomy status.

#### **Appendix Table 4**. Structural brain differences between HT users vs. non-users

| Contrast | Ke | MNI coordinates | | | Z | | P_FWE-corr_* | P_uncorr_ | Side | | Region | |  |
| --- | --- | --- | --- | --- | --- | --- | --- | --- | --- | --- | --- | --- | --- |
|  |  | x | y | z |  |  | |  | |  | |  | |
| **Gray matter volume** | | | | | | | | | | | | |  |
| Users >non-users | 50 | 54 | -3 | -40 | 4.03 | 0.023 | | <0.001 | | Right | | Inferior temporal gyrus | |
| Non-users >users | n.s. |  |  |  |  |  | |  | |  | |  | |
| **White matter volume** | | | | | | | | | | | | |  |
| Users >non-users | n.s. |  |  |  |  |  | |  | |  | |  | |
| Non-users >users | n.s. |  |  |  |  |  | |  | |  | |  | |
| **Fractional anisotropy** | | | | | | | | | | | | |  |
| Users >non-users | n.s. |  |  |  |  |  | |  | |  | |  | |
| Non-users >users | n.s. |  |  |  |  |  | |  | |  | |  | |

*p<0.05 gray matter-masked (gray matter) or white-matter masked (white matter volume and fractional anisotropy), cluster-level corrected for Family-Type Wise Error (FWE), adjusted by total intracranial volume. Abbreviations: Ke, cluster extent (voxels).

### Metabolic measures

CMRglc and CBF results are reported in **Appendix Table 5**. Adjusting for age and global uptake, HT non-users showed lower CMRglc in middle temporal gyrus as compared to HT users. There were no group differences for CBF measures. Results remained unchanged adjusting for hysterectomy status.

#### **Appendix Table 5**. Metabolic differences between HT users vs. non-users

| Contrast | Ke | MNI coordinates | | | | Z | P_FWE-corr_* | | | P_uncorr_ | Side | | | Region |  |
| --- | --- | --- | --- | --- | --- | --- | --- | --- | --- | --- | --- | --- | --- | --- | --- |
|  |  | x | y | z |  | | |  |  | | |  |  | | |
| **CMRglc** | | | | | | | | | | | | | | |  |
| Users >non-users | 624 | -57 | -69 | 14 | 4.07 | | | 0.004 | <0.001 | | | Left | Middle temporal gyrus | | |
|  |  | -51 | -75 | 18 | 3.50 | | |  | <0.001 | | | Left | Middle temporal gyrus | | |
| Non-users >users | n.s. |  |  |  |  | | |  |  | | |  |  | | |
| **Cerebral Blood Flow** | | | | | | | | | | | | | | |  |
| Users >non-users | n.s. |  |  |  |  | | |  |  | | |  |  | | |
| Non-users >users | n.s. |  |  |  |  | | |  |  | | |  |  | | |

*p<0.05 gray matter-masked, cluster-level corrected for Family-Type Wise Error (FWE), adjusted by global activity. Abbreviations: Ke, cluster extent (voxels).

There were no significant differences in ATP/PCr measures between HT users and non-users (**Appendix Table 6**).

#### **Appendix Table 6**. MRS ATP/PCr measures by HT group

|  | **HT non-users** | **HT users** |
| --- | --- | --- |
| N | 21 | 24 |
| Superior temporal gyrus | 1.23(0.03) | 1.26(0.03) |
| *adjusted by hysterectomy status* | 1.24(0.03) | 1.26(0.03) |
| Middle temporal gyrus | 1.18(0.03) | 1.21(0.03) |
| *adjusted by hysterectomy status* | 1.18(0.03) | 1.20(0.03) |
| Inferior temporal gyrus | 1.14(0.03) | 1.15(0.03) |
| *adjusted by hysterectomy status* | 1.13(0.03) | 1.15(0.03) |
| Inferior parietal lobule | 0.84(0.03) | 0.80(0.03) |
| *adjusted by hysterectomy status* | 0.84(0.03) | 0.80(0.03) |

Values are age-adjusted means (SE).

### Amyloid-β load

Results are shown in **Appendix Table 7.** Adjusting for age and hysterectomy status, there were no significant differences in PiB uptake between HT users and non-users. Descriptively, HT non-users had 11% higher PiB uptake than HT users, and included 18% more amyloid-positive scans, which did not reach significance.

#### **Appendix Table 7**. PiB-PET amyloid-β load by post-menopause HT group

|  | **HT non-users** | **HT users** |
| --- | --- | --- |
| N | 30 | 17 |
| AD-mask SUVR | 1.34(0.08) | 1.21(0.10) |
| *adjusted by hysterectomy status* | 1.34(0.08) | 1.20(0.11) |
| PiB scans, % positive | 30 | 12 |

Values are age-adjusted means (SE), unless otherwise specified. Abbreviations: SUVR, standardized uptake value ratio to cerebellar gray matter PiB uptake.

## Biomarker differences between MT groups among HT non-users

Given above evidence of some biomarker differences between HT users and non-users, we repeated all analyses of menopausal group differences among HT non-users. Groups were comparable for demographical characteristics except for age (**Appendix Table 8**).

#### **Table 8**. Participants’ characteristics by MT status among HT non-users

|  | **PRE** | **PERI** | **POST** |
| --- | --- | --- | --- |
| N | 30 | 44 | 41 |
| Age, y, range | 44(4) | 50(4) | 57(4) |
| Education, y | 17(2) | 17(2) | 17(2) |
| Ethnicity, % White | 80 | 75 | 90 |
| MMSE score | 29(1) | 29(1) | 29(1) |
| *APOE* ε4 carriers, % positive | 53 | 23 | 49 |
| Hysterectomy status, % positive | 0 | 5 | 15 |

Values are means (SD), unless otherwise specified.

### Structural measures

Results are reported in **Appendix Table 9**. Among HT non-users, menopausal group differences in GM and WM volumes were consistent with those observed in the entire cohort. Findings of lower FA in external capsule of the post-menopausal group vs. the peri-menopausal group were not observed among HT non-users.

#### **Appendix Table 9**. Structural group differences among HT non-users

| Contrast | Ke | MNI coordinates | | | Z | | P_FWE-corr_* | P_uncorr_ | | Side | | Region |  |
| --- | --- | --- | --- | --- | --- | --- | --- | --- | --- | --- | --- | --- | --- |
|  |  | x | y | z |  |  | |  |  | |  | | |
| **Gray matter volume** | | | | | | | | | | | | |  |
| POST<PRE | 52 | 48 | -50 | -24 | 3.33 | 0.019 | | <0.001 | Right | | Inferior temporal gyrus | | |
| POST>PERI | 38 | 28 | -80 | -6 | 2.64 | 0.039 | | <0.001 | Right | | Fusiform gyrus | | |
|  | *20* | *20* | *-58* | *30* | *3.62* | *0.111* | | *0.004* | *Right* | | *Precuneus* | | |
| **White matter volume** | | | | | | | | | | | | |  |
| POST<PRE | 132 | 35 | -39 | 26 | 3.05 | 0.031 | | <0.001 | Right | | Superior longitudinal fasciculus | | |
|  | 165 | -33 | -39 | 27 | 3.10 | 0.028 | | <0.001 | Left | | Superior longitudinal fasciculus | | |
|  | 112 | -33 | -1 | 25 | 3.04 | 0.032 | | <0.001 | Left | | Superior longitudinal fasciculus | | |
| POST<PERI | 545 | 33 | 9 | 21 | 3.57 | 0.011 | | <0.001 | Right | | Superior longitudinal fasciculus | | |
|  |  | 34 | -16 | 33 | 3.08 |  | | 0.001 | Right | | Superior longitudinal fasciculus | | |
|  | 250 | -36 | -17 | 25 | 3.23 | 0.028 | | <0.001 | Left | | Superior longitudinal fasciculus | | |
|  | 167 | -20 | -17 | -8 | 3.51 | 0.012 | | <0.001 | Left | | Cerebral peduncle | | |
| **Fractional anisotropy** | | | | | | | | | | | | |  |
| POST<PRE | n.s. |  |  |  |  |  | |  |  | |  | | |
| POST<PERI | n.s. |  |  |  |  |  | |  |  | |  | | |

*p<0.05 gray matter-masked (gray matter) or white-matter masked (white matter volume and fractional anisotropy), cluster-level corrected for Family-Type Wise Error (FWE), adjusted by total intracranial volume. Only significant contrasts are reported. Abbreviations: Ke, cluster extent (voxels).

### Metabolic measures

Results are reported in **Appendix Table 10**. Among HT non-users, MT group differences in CMRglc and CBF measures were consistent with those observed in the entire cohort. The POST group showed lower CMRglc in temporo-parietal regions than the other groups, and the PERI group also showed lower temporal CMRglc than the PRE group. The POST group showed higher CBF in temporo-parietal regions than both other groups, whereas in the main analysis the differences between POST and PRE groups did not reach significance.

#### **Appendix Table 10**. Metabolic group differences among HT non-users

| Contrast | Ke | MNI coordinates | | | | Z | P_FWE-corr_* | | | P_uncorr_ | | Side | | Region |  |
| --- | --- | --- | --- | --- | --- | --- | --- | --- | --- | --- | --- | --- | --- | --- | --- |
|  |  | x | y | z |  | | |  |  | |  | |  | | |
| **CMRglc** | | | | | | | | | | | | | | |  |
| POST<PRE | 67 | 56 | -38 | 2 | 3.35 | | | 0.035 | <0.001 | | Right | | Middle temporal gyrus | | |
|  | 129 | -58 | -51 | 9 | 3.48 | | | 0.050 | <0.001 | | Left | | Middle temporal gyrus | | |
|  |  | -54 | -58 | 18 | 2.89 | | |  | <0.001 | | Left | | Angular gyrus | | |
| POST<PERI | 35 | -58 | -52 | 10 | 3.75 | | | 0.013 | <0.001 | | Left | | Middle temporal gyrus | | |
|  | 64 | 8 | -48 | 68 | 3.06 | | | 0.036 | <0.001 | | Right | | Precuneus | | |
| PERI<PRE | 46 | -58 | -52 | 10 | 3.57 | | | 0.026 | <0.001 | | Left | | Middle temporal gyrus | | |
| **Cerebral blood flow** | | | | | | | | | | | | | | |  |
| POST>PRE | 90 | -24 | -4 | 46 | 3.60 | | | 0.011 | <.001 | | Left | | Superior frontal gyrus | | |
|  | 349 | -63 | -51 | -10 | 3.30 | | | 0.026 | <.001 | | Left | | Middle temporal gyrus | | |
|  | 68 | 58 | -14 | -26 | 3.03 | | | 0.050 | .001 | | Right | | Middle temporal gyrus | | |
| POST>PERI | 183 | -51 | -24 | 34 | 3.48 | | | 0.040 | <.001 | | Left | | Supramarginal gyrus | | |
|  | 231 | -66 | -6 | -21 | 3.43 | | | 0.050 | <.001 | | Left | | Middle temporal gyrus | | |
|  | 165 | 56 | -32 | 6 | 3.24 | | | 0.031 | <.001 | | Left | | Superior temporal gyrus | | |

*p<0.05 gray matter-masked, cluster-level corrected for Family-Type Wise Error (FWE), adjusted by global activity. Only significant contrasts are reported. Abbreviations: Ke, cluster extent (voxels).

As in the main analysis, we examined ATP/PCr measures in the superior, middle, inferior temporal cortex, and inferior parietal lobule. General linear models showed higher ATP/PCr in the POST group compared to the PRE group in inferior, middle, and superior temporal gyrus (p’s<0.042), and intermediate levels in the PERI group, which did not reach significance (**Appendix Table 11**).

#### **Appendix Table 11**. MRS ATP/PCr measures by menopause group within the group of HT non-users

|  | **PRE** | **PERI** | **POST** |
| --- | --- | --- | --- |
| N | 16 | 24 | 21 |
| Superior temporal gyrus | 1.14(0.03) | 1.16(0.03) | 1.23(0.03)* |
| Middle temporal gyrus | 1.10(0.03) | 1.13(0.02) | 1.18(0.02)* |
| Inferior temporal gyrus | 1.07(0.03) | 1.11(0.02) | 1.14(0.02)* |
| Inferior parietal lobule | 0.77(0.05) | 0.79(0.04) | 0.84(0.04) |

Values are means (SE). *Different from the PRE group on univariate post-hoc analysis, p<0.05.

### Amyloid-β load

PiB uptake measures and PiB-positive scan data are reported in **Appendix Table 12**. There were significant difference in PiB AD-mask uptake between MT groups (p=0.030), which on post-hoc examination were driven by the POST group showing higher PiB uptake than the PRE group (p=0.008), and borderline higher compared to the PERI group (p=0.110). Results were unchanged by adding APOE-4 status as a covariate (overall p=0.038).

The APOE group also included a higher frequency of PiB-positive cases as compared to the PRE group (30% vs. 5%, respectively), while the PERI group fell in between (15%).

#### **Appendix Table 12**. PiB-PET amyloid-β load by menopause group among HT non-users

|  | **PRE** | **PERI** | **POST** |
| --- | --- | --- | --- |
| N | 21 | 34 | 30 |
| AD-mask SUVR | 1.00(0.09) | 1.20(0.07) | 1.34(0.08)* |
| *adjusted by APOE status* | 1.01(0.09) | 1.20(0.07) | 1.33(0.08)* |
| PiB scans, % positive | 5 | 15 | 30* |

Values are age-adjusted means (SE), unless otherwise specified. *Different from the PRE group, p<0.05. Abbreviations: SUVR, standardized uptake value ratio to cerebellar gray matter PiB uptake.

## Conclusions

Overall, MT-related brain biomarker effects were independent of HT use and hysterectomy status, and MT stage effects among HT non-users are largely consistent with those observed in the full dataset.
