# Supplementary material for: Menopause impacts human brain structure, connectivity, energy metabolism, and amyloid-beta deposition
Source: Sci Rep. 2021 Jun 9;11:10867. doi: 10.1038/s41598-021-90084-y (PMC8190071; doi:10.1038/s41598-021-90084-y)
Supplement: Supplementary file 2 — Supplementary Information 2. [file 41598_2021_90084_MOESM2_ESM.docx]

# Menopause impacts human brain structure, connectivity,

# energy metabolism, and amyloid-beta deposition

Lisa Mosconi, PhD^1,2,3*^, Valentina Berti, MD, PhD^4^, Jonathan Dyke, PhD^2^, Eva Schelbaum, MA^1^, Steven Jett, BA^1^, Lacey Loughlin, BA^1^, Grace Jang, BA^1^, Aneela Rahman, PhDc^1^, Hollie Hristov, NP^1^, Silky Pahlajani, MD^1,2^, Randolph Andrews, BS^5^, Dawn Matthews, PhD^5^, Orli Etingin, MD^6^, Christine Ganzer, PhD^7^, Mony de Leon, EdD^2^, Richard Isaacson, MD^1^, Roberta Diaz Brinton, PhD^8^.

^1^ Department of Neurology, Weill Cornell Medical College, New York NY, USA

^2^ Department of Radiology, Weill Cornell Medical College, New York NY, USA

^3^ Department of Psychiatry, New York University School of Medicine, New York NY, USA

^4^ Department of Nuclear Medicine, University of Florence, Italy

^5^ ADM Diagnostics, Chicago, IL

^6^ Department of Internal Medicine, Weill Cornell Medical College, New York NY, USA

^7^ Hunter-Bellevue School of Nursing, Hunter College, CUNY, New York NY, USA

^8^ Departments of Pharmacology and Neurology, College of Medicine, University of Arizona, Tucson, AZ, USA

## **Supplementary Table S1**. Participant characteristics of male groups

|  | **MALE_PRE_** | **MALE_PERI_** | **MALE_POST_** |
| --- | --- | --- | --- |
| N | 30 | 50 | 45 |
| Age, years, range | 45(4), 40-53 | 50(6), 41-60 | 56(6), 46-65 |
| Education, years | 17(2) | 17(2) | 17(3) |
| Ethnicity, % White | 70 | 70 | 71 |
| MMSE scores | 29(1) | 29(1) | 29(1) |
| APOE ε4 carriers, % positive | 43 | 44 | 42 |

Values are means (SD), unless otherwise specified.

Abbreviations: MALE_PRE,_ males age-matched to PRE; MALE_PERI,_ males age-matched to PERI; MALE_POST,_ males age-matched to POST.

## **Supplementary Table S2**. Gray and white matter volume differences between menopause groups

| **Contrast** | | **Ke** | **MNI coordinates** | | | | | | | **Z** | | **P_FWE_*** | | **P_uncorr_** | | **Side** | | **Region** | |
| --- | --- | --- | --- | --- | --- | --- | --- | --- | --- | --- | --- | --- | --- | --- | --- | --- | --- | --- | --- |
|  | |  | x | | | y | | z | |  | |  | |  | |  | |  | |
| **Gray matter volume** | | | | | | | | | | | | | | | | | | | |
| POST<PRE | | 284 | 46 | | | -52 | | -22 | | 3.51 | | 0.016 | | <0.001 | | Right | | Inferior temporal gyrus | |
| POST>PRE | | -- |  | | |  | |  | |  | |  | |  | |  | |  | |
| POST<PERI | | -- |  | | |  | |  | |  | |  | |  | |  | |  | |
| POST>PERI | | 251 | 20 | | | -58 | | 30 | | 3.58 | | 0.014 | | <0.001 | | Right | | Precuneus | |
|  | | 231 | 30 | | | -76 | | -4 | | 3.35 | | 0.014 | | <0.001 | | Right | | Fusiform gyrus | |
|  | | *101* | *-12* | | | *-62* | | *26* | | *3.19* | | *0.057* | | *<0.001* | | *Left* | | *Precuneus* | |
| PERI<PRE | | -- |  | | |  | |  | |  | |  | |  | |  | |  | |
| PERI>PRE | | -- |  | | |  | |  | |  | |  | |  | |  | |  | |
| **White matter volume** | | | | | | | | | | | | | | | | | | |  |
| POST<PRE | 43 | | | 28 | -51 | | 27 | | 3.42 | | 0.008 | | <0.001 | | Right | | Posterior corona radiata | |  |
| POST>PRE | -- | | |  |  | |  | |  | |  | |  | |  | |  | |  |
| POST<PERI | 93 | | | 20 | -51 | | 30 | | 3.68 | | 0.003 | | <0.001 | | Right | | Posterior corona radiata | |  |
|  | 52 | | | 28 | 14 | | 17 | | 3.42 | | 0.008 | | <0.001 | | Right | | Anterior corona radiata | |  |
| POST>PERI | -- | | |  |  | |  | |  | |  | |  | |  | |  | |  |
| PERI<PRE | -- | | |  |  | |  | |  | |  | |  | |  | |  | |  |
| PERI>PRE | -- | | |  |  | |  | |  | |  | |  | |  | |  | |  |

*p<0.05 cluster-level corrected for Family-Type Wise Error (FWE), adjusted by age and total intracranial volume. The cluster showing borderline significance is reported in italics.

Abbreviations: Ke, cluster extent (voxels); PERI, peri-menopausal group; POST, post-menopausal group; PRE, pre-menopausal group.

## **Supplementary Table S3**. Brain metabolic differences between menopause groups.

| **Contrast** | | **Ke** | | **MNI coordinates** | | | | | **Z** | | **P_FWE_*** | | **P_uncorr_** | | **Side** | | **Region** | | |
| --- | --- | --- | --- | --- | --- | --- | --- | --- | --- | --- | --- | --- | --- | --- | --- | --- | --- | --- | --- |
|  | |  | | x | | y | z | |  | |  | |  | |  | |  | | |
| **Cerebral glucose metabolism** | | | | | | | | | | | | | | | | | | | |
| POST<PRE | | 583 | | 56 | | -38 | 2 | | 3.73 | | 0.010 | | <0.001 | | Right | | Middle temporal gyrus | | |
|  | | 1199 | | -58 | | -51 | 9 | | 3.68 | | 0.012 | | <0.001 | | Left | | Middle temporal gyrus | | |
|  | |  | | -57 | | -52 | -2 | | 3.13 | |  | | <0.001 | | Left | | Middle temporal gyrus | | |
|  | | 255 | | -54 | | -45 | 26 | | 3.52 | | 0.021 | | <0.001 | | Left | | Supramarginal gyrus | | |
|  | | 376 | | 51 | | -46 | -24 | | 3.29 | | 0.025 | | <0.001 | | Right | | Inferior temporal gyrus | | |
| POST>PRE | | -- | |  | |  |  | |  | |  | |  | |  | |  | | |
| POST<PERI | | 879 | | -58 | | -52 | 10 | | 3.73 | | 0.018 | | <0.001 | | Left | | Middle temporal gyrus | | |
|  | |  | | -58 | | -54 | -10 | | 3.18 | |  | | <0.001 | | Left | | Inferior temporal gyrus | | |
| POST>PERI | | -- | |  | |  |  | |  | |  | |  | |  | |  | | |
| PERI<PRE | | 498 | | 56 | | -39 | 4 | | 3.44 | | 0.020 | | <0.001 | | Right | | Middle temporal gyrus | | |
| PERI>PRE | | -- | |  | |  |  | |  | |  | |  | |  | |  | | |
| **Cerebral blood flow** | | | | | | | | | | | | | | | | | | |  |
| POST<PRE | -- | |  | |  | | |  | |  | |  | |  | |  | |  |  |
| POST>PRE | -- | |  | |  | | |  | |  | |  | |  | |  | |  |  |
| POST<PERI | -- | |  | |  | | |  | |  | |  | |  | |  | |  |  |
| POST>PERI | 670 | | 62 | | -33 | | | 27 | | 3.96 | | 0.014 | | <.001 | | Right | | Supramarginal gyrus |  |
|  |  | | 56 | | -32 | | | 6 | | 3.51 | |  | | <.001 | | Right | | Superior temporal gyrus |  |
|  |  | | 64 | | -28 | | | -4 | | 3.15 | |  | | <.001 | | Right | | Middle temporal gyrus |  |
|  | 122 | | 14 | | 21 | | | 56 | | 3.58 | | 0.038 | | <.001 | | Right | | Superior frontal gyrus |  |
|  | 166 | | -54 | | -28 | | | 27 | | 3.46 | | 0.033 | | <.001 | | Left | | Supramarginal gyrus |  |
|  | 472 | | 51 | | -14 | | | -2 | | 3.44 | | 0.025 | | <.001 | | Right | | Superior temporal gyrus |  |
|  | 256 | | -39 | | 32 | | | 15 | | 3.42 | | 0.024 | | <.001 | | Left | | Inferior frontal gyrus |  |
|  | 289 | | -64 | | -8 | | | -24 | | 3.40 | | 0.029 | | <.001 | | Left | | Middle temporal gyrus |  |
| PERI<PRE | -- | |  | |  | | |  | |  | |  | |  | |  | |  |  |
| PERI>PRE | -- | |  | |  | | |  | |  | |  | |  | |  | |  |  |

*p<0.05 cluster-level corrected for FWE, adjusted by age and global activity.

Abbreviations: see legend to Supplementary Table S3.

## **Supplementary Table S4**. Gray matter volume differences between menopausal groups and age-matched males.

| **Contrast** | **Ke** | **MNI coordinates** | | | | **Z** | **P_FWE_*** | **P_uncorr_** | **Side** | | | **Region** |  |
| --- | --- | --- | --- | --- | --- | --- | --- | --- | --- | --- | --- | --- | --- |
|  |  | x | y | z |  | |  |  | |  |  | | |
| POST<MALE_POST_ | 10088 | -26 | -4 | -22 | 10.0 | | <0.001 | <0.001 | | Left | Hippocampus | | |
|  |  | -22 | -54 | -14 | 9.67 | |  | <0.001 | | Left | Fusiform gyrus | | |
|  | 8614 | 26 | -51 | -14 | 9.63 | | <0.001 | <0.001 | | Right | Fusiform gyrus | | |
|  |  | 26 | -3 | -22 | 9.33 | |  | <0.001 | | Right | Hippocampus | | |
|  |  | 27 | -2 | -20 | 9.27 | |  | <0.001 | | Right | Amygdala | | |
|  | 932 | -4 | 32 | -7 | 5.76 | | <0.001 | <0.001 | | Left | Anterior cingulate gyrus | | |
|  | 442 | 10 | -24 | 6 | 5.85 | | <0.001 | <0.001 | | Right | Thalamus | | |
|  | 2019 | -36 | 24 | 3 | 5.84 | | <0.001 | <0.001 | | Left | Insula | | |
|  |  | -39 | 0 | 2 | 5.83 | |  | <0.001 | | Left | Insula | | |
|  |  | -48 | 6 | -10 | 5.73 | |  | <0.001 | | Left | Temporal pole | | |
|  | 468 | 24 | 62 | 16 | 5.47 | | <0.001 | <0.001 | | Right | Superior frontal gyrus | | |
|  |  | 16 | 52 | 36 | 4.81 | |  | <0.001 | | Right | Superior frontal gyrus | | |
|  | 122 | -16 | 56 | 30 | 5.29 | | <0.001 | <0.001 | | Left | Superior frontal gyrus | | |
|  | 307 | 60 | -6 | -16 | 5.17 | | <0.001 | <0.001 | | Right | Middle temporal gyrus | | |
|  |  | 57 | 0 | -21 | 5.08 | |  | <0.001 | | Right | Middle temporal gyrus | | |
|  | 243 | -12 | -27 | 6 | 5.14 | | <0.001 | <0.001 | | Left | Thalamus | | |
|  |  | -33 | 2 | -3 | 5.08 | |  | <0.001 | | Left | Putamen | | |
|  | 115 | 30 | 36 | 42 | 5.04 | | 0.006 | <0.001 | | Right | Middle frontal gyrus | | |
|  |  | -12 | 15 | -20 | 4.99 | |  | <0.001 | | Left | Orbitofrontal gyrus | | |
|  | 80 | 56 | -33 | 50 | 4.90 | | 0.01 | <0.001 | | Right | Supramarginal gyrus | | |
|  | 505 | -4 | 33 | -10 | 4.89 | | 0.005 | <0.001 | | Left | Orbitofrontal gyrus | | |
|  | 287 | -58 | -42 | 42 | 4.87 | | 0.001 | <0.001 | | Left | Supramarginal gyrus | | |
|  |  | -60 | -32 | 40 | 4.49 | |  | <0.001 | | Left | Supramarginal gyrus | | |
|  | 57 | 45 | 46 | -2 | 4.83 | | 0.01 | <0.001 | | Right | Orbitofrontal gyrus | | |
|  | 52 | 63 | -40 | 18 | 4.73 | | 0.01 | <0.001 | | Right | Superior temporal gyrus | | |
|  | 121 | 60 | -42 | -12 | 4.68 | | 0.004 | <0.001 | | Right | Inferior temporal gyrus | | |
|  | 122 | -56 | -8 | -14 | 4.65 | | 0.004 | <0.001 | | Left | Middle temporal gyrus | | |
|  |  | -58 | -18 | -9 | 4.60 | |  | <0.001 | | Left | Middle temporal gyrus | | |
|  | 262 | -6 | -44 | 31 | 4.63 | | 0.007 | <0.001 | | Left | Posterior cingulate gyrus | | |
|  | 108 | -54 | -57 | 20 | 4.52 | | 0.01 | <0.001 | | Left | Middle temporal gyrus | | |
| POST>MALE_POST_ | -- |  |  |  |  | |  |  | |  |  | | |
| PERI<MALE_PERI_ | 16391 | -28 | -44 | -18 | 7.34 | | <0.001 | <0.001 | | Left | Fusiform gyrus | | |
|  |  | -28 | -2 | -42 | 6.83 | |  | <0.001 | | Right | Inferior temporal gyrus | | |
|  |  | -27 | -4 | -22 | 6.88 | |  | <0.001 | | Left | Hippocampus | | |
|  |  | -58 | -18 | -9 | 4.60 | |  | <0.001 | | Left | Middle temporal gyrus | | |
|  | 15269 | 26 | -48 | -18 | 7.29 | | <0.001 | <0.001 | | Right | Fusiform gyrus | | |
|  |  | 28 | 0 | -21 | 6.76 | |  | <0.001 | | Right | Entorhinal area | | |
|  |  | 32 | -8 | -40 | 6.44 | |  | <0.001 | | Right | Fusiform gyrus | | |
|  | 1007 | 46 | 30 | -2 | 5.94 | | <0.001 | <0.001 | | Right | Orbitofrontal gyrus | | |
|  | 4488 | 12 | -51 | 10 | 5.38 | | <0.001 | <0.001 | | Right | Lingual gyrus | | |
|  |  | 6 | -54 | 20 | 5.27 | |  | <0.001 | | Right | Precuneus | | |
|  |  | -8 | -60 | 15 | 4.92 | |  | <0.001 | | Left | Precuneus | | |
|  | 556 | -33 | 2 | -2 | 3.68 | | <0.001 | <0.001 | | Left | Putamen | | |
|  |  | -33 | -15 | -4 | 3.36 | |  | <0.001 | | Left | Putamen | | |
|  |  | -33 | -22 | -6 | 3.36 | |  | <0.001 | | Left | Insula | | |
|  | 190 | -18 | 58 | 24 | 4.96 | | <0.001 | <0.001 | | Left | Superior frontal gyrus | | |
|  |  | -8 | 38 | -6 | 4.93 | |  | <0.001 | | Left | Anterior cingulate gyrus | | |
|  | 88 | -28 | 15 | 52 | 4.08 | | 0.005 | <0.001 | | Right | Middle frontal gyrus | | |
|  | 67 | -42 | 26 | 24 | 3.79 | | 0.005 | <0.001 | | Left | Inferior frontal gyrus | | |
|  | 123 | 28 | 34 | 40 | 3.50 | | 0.003 | <0.001 | | Right | Middle frontal gyrus | | |
|  |  | 33 | 0 | 54 | 3.45 | |  | <0.001 | | Right | Middle frontal gyrus | | |
| PERI>MALE_PERI_ | -- |  |  |  |  | |  |  | |  |  | | |
| PRE<MALE_PRE_ | 3346 | 27 | -2 | -24 | 4.92 | | <0.001 | <0.001 | | Right | Amygdala | | |
|  |  | 34 | 8 | -2 | 4.42 | |  | <0.001 | | Right | Putamen | | |
|  |  | 48 | 21 | 12 | 4.39 | |  | <0.001 | | Right | Inferior frontal gyrus | | |
|  | 1936 | -24 | -6 | -24 | 4.75 | | <0.001 | <0.001 | | Left | Hippocampus | | |
|  |  | -48 | 0 | -36 | 4.22 | |  | <0.001 | | Left | Inferior temporal gyrus | | |
|  |  | -27 | 2 | -44 | 4.07 | |  | <0.001 | | Left | Fusiform gyrus | | |
|  | 874 | -45 | -40 | -22 | 4.57 | | 0.001 | <0.001 | | Left | Inferior temporal gyrus | | |
|  |  | -24 | -57 | -16 | 4.08 | |  | <0.001 | | Left | Superior frontal gyrus | | |
|  | 335 | -12 | -16 | 3 | 4.33 | | 0.002 | <0.001 | | Left | Thalamus | | |
|  | 563 | -45 | 21 | 8 | 4.21 | | 0.002 | <0.001 | | Left | Inferior frontal gyrus | | |
|  |  | -37 | 20 | 0 | 3.58 | |  | <0.001 | | Left | Insula | | |
|  | 144 | 34 | 44 | -10 | 3.96 | | 0.007 | <0.001 | | Right | Orbitofrontal gyrus | | |
|  | 85 | 21 | 16 | -18 | 3.86 | | 0.011 | <0.001 | | Right | Orbitofrontal gyrus | | |
|  | 210 | -54 | -4 | -14 | 3.78 | | 0.004 | <0.001 | | Left | Middle temporal gyrus | | |
|  | 80 | -50 | -62 | -10 | 3.59 | | 0.012 | <0.001 | | Left | Inferior temporal gyrus | | |
| PRE>MALE_PRE_ | -- |  |  |  |  | |  |  | |  |  | | |

*p<0.05, cluster-level corrected for Family-Type Wise Error (FWE), adjusted by age and total intracranial volume.

Abbreviations: Ke, cluster extent (voxels); MALE_PRE,_ males age-matched to PRE; MALE_PERI,_ males age-matched to PERI; MALE_POST,_ males age-matched to POST; PERI, peri-menopausal group; POST, post-menopausal group; PRE, pre-menopausal group.

## **Supplementary Table S5**. White matter volume differences between menopausal groups and age-matched males.

| **Contrast** | **Ke** | **MNI coordinates** | | | **Z** | **P_FWE_*** | **P_uncorr_** | **Side** | **Region** |
| --- | --- | --- | --- | --- | --- | --- | --- | --- | --- |
|  |  | x | y | z |  |  |  |  |  |
| POST<MALE_POST_ | 42610 | 38 | -22 | 29 | 6.14 | <0.001 | <0.001 | Right | Superior longitudinal fasciculus |
|  |  | 19 | 2 | 37 | 5.62 |  | <0.001 | Right | Superior corona radiata |
|  |  | 19 | 37 | 5 | 5.49 |  | <0.001 | Right | Anterior corona radiata |
|  |  | 14 | -11 | 0 | 5.47 |  | <0.001 | Right | Posterior limb of internal capsule |
|  |  | 16 | -15 | -5 | 4.92 |  | <0.001 | Right | Cerebral peduncle |
|  |  | 38 | -43 | 5 | 4.48 |  | <0.001 | Right | Posterior thalamic radiation |
|  |  | 14 | -37 | 29 | 4.01 |  | <0.001 | Right | Splenium of Corpus callosum |
|  | 28300 | -6 | -30 | -30 | 8.28 | <0.001 | <0.001 | Left | Pontine crossing tract |
|  |  | 4 | -30 | -30 | 6.71 | <0.001 | <0.001 | Right | Pontine crossing tract |
|  |  | 6 | -25 | -33 | 5.76 | <0.001 | <0.001 | Right | Cortico-spinal tract |
|  |  | -8 | -25 | -31 | 7.22 | <0.001 | <0.001 | Left | Cortico-spinal tract |
|  | 54619 | -34 | -33 | 29 | 6.85 | <0.001 | <0.001 | Left | Superior longitudinal fasciculus |
|  |  | -26 | -5 | 29 | 5.62 |  | <0.001 | Left | Superior corona radiata |
|  |  | -26 | 17 | 22 | 5.61 |  | <0.001 | Left | Anterior corona radiata |
|  |  | -17 | -4 | 9 | 5.83 |  | <0.001 | Left | Posterior limb of internal capsule |
|  |  | -35 | 29 | 6 | 4.22 |  | <0.001 | Left | Retrolenticular part of internal capsule |
|  |  | -35 | -61 | 9 | 4.32 |  | <0.001 | Left | Posterior thalamic radiation |
|  |  | -11 | -39 | 29 | 3.83 |  | <0.001 | Left | Cingulate gyrus |
| POST>MALE_POST_ | -- |  |  |  |  |  |  |  |  |
| PERI<MALE_PERI_ | 14888 | 19 | -12 | -4 | 4.79 | <0.001 | <0.001 | Right | Cerebral peduncle / Posterior limb of internal capsule |
|  |  | 20 | -39 | 34 | 4.84 |  | <0.001 | Right | Posterior corona radiata |
|  |  | 32 | -23 | 34 | 5.33 |  | <0.001 | Right | Superior longitudinal fasciculus |
|  |  | 27 | -13 | 27 | 5.18 |  | <0.001 | Right | Superior corona radiata |
|  |  | 6 | -24 | -30 | 5.65 |  | <0.001 | Right | Cortico-spinal tract |
|  |  | 8 | -31 | -31 | 5.73 |  | <0.001 | Right | Pontine crossing tract |
|  | 12714 | -24 | -18 | 12 | 5.14 | <0.001 | <0.001 | Left | Posterior limb of internal capsule |
|  |  | -24 | -31 | 35 | 5.48 |  | <0.001 | Left | Posterior corona radiata |
|  |  | -36 | -45 | 25 | 5.60 |  | <0.001 | Left | Superior longitudinal fasciculus |
|  |  | -24 | -15 | 34 | 5.38 |  | <0.001 | Left | Superior corona radiata |
|  |  | -8 | -28 | -32 | 6.01 |  | <0.001 | Left | Cortico-spinal tract |
| PERI>MALE_PERI_ | -- |  |  |  |  |  |  |  |  |
| PRE<MALE_PRE_ | 1103 | -14 | -7 | 1 | 5.28 | <0.001 | <0.001 | Left | Posterior limb of internal capsule |
|  | 1841 | 15 | -9 | 2 | 4.92 | <0.001 | <0.001 | Right | Posterior limb of internal capsule |
| PRE>MALE_PRE_ | -- |  |  |  |  |  |  |  |  |

*p<0.05, cluster-level corrected, adjusted by age and total intracranial volume.

See legend to Supplementary Table S5.

## **Supplementary Table S6**. Fractional anisotropy differences between menopausal groups and age-matched males.

| **Contrast** | **Ke** | **MNI coordinates** | | | **Z** | **P_FWE_*** | **P_uncorr_** | **Side** | **Region** |
| --- | --- | --- | --- | --- | --- | --- | --- | --- | --- |
|  |  | x | y | z |  |  |  |  |  |
| POST<MALE_POST_ | -- |  |  |  |  |  |  |  |  |
|  |  |  |  |  |  |  |  |  |  |
| POST>MALE_POST_ | 198 | -26 | 23 | 7 | 3.93 | 0.015 | <0.001 | Left | Anterior corona radiata |
|  | 56 | -30 | -17 | -10 | 3.82 | 0.021 | <0.001 | Left | Fornix, Stria terminalis |
| PERI<MALE_PERI_ | 53 | -38 | -3 | 23 | 3.84 | 0.022 | <0.001 | Left | Superior longitudinal fasciculus |
|  | 132 | -40 | -38 | 15 | 3.58 | 0.050 | <0.001 | Left | Posterior thalamic radiation |
| PERI>MALE_PERI_ | 491 | -34 | -16 | -13 | 4.20 | 0.006 | <0.001 | Left | Fornix, Stria terminalis |
|  |  | -32 | 2 | -15 | 4.01 |  | <0.001 | Left | Uncinate fasciculus |
|  | 120 | -3 | 5 | 15 | 4.17 | 0.020 | <0.001 | Left | External capsule |
|  | 189 | 21 | -49 | 32 | 4.14 | 0.022 | <0.001 | Right | Posterior corona radiata |
| PRE<MALE_PRE_ | 52 | -42 | -45 | 13 | 3.68 | 0.050 | <0.001 | Left | Superior longitudinal fasciculus |
| PRE>MALE_PRE_ | 71 | 22 | -31 | 38 | 4.28 | 0.039 | <0.001 | Right | Posterior corona radiata |

*p<0.05, cluster-level corrected, adjusted by age and total intracranial volume.

Abbreviations: see legend to Supplementary Table S5.

## **Supplementary Table S7**. FDG glucose metabolism differences between menopausal groups and age-matched males.

| **Contrast** | **Ke** | **MNI coordinates** | | | **Z** | **P_FWE_*** | **P_uncorr_** | **Side** | **Region** |
| --- | --- | --- | --- | --- | --- | --- | --- | --- | --- |
|  |  | x | y | z |  |  |  |  |  |
| POST<MALE_POST_ | 1088 | -24 | 58 | 27 | 4.02 | 0.004 | <0.001 | Left | Middle frontal gyrus |
|  |  | -14 | 27 | 60 | 3.95 |  | <0.001 | Left | Superior frontal gyrus |
|  | 410 | 16 | 52 | 36 | 3.82 | 0.005 | <0.001 | Right | Superior frontal gyrus |
|  |  | 22 | 40 | 51 | 3.52 |  | <0.001 | Right | Superior frontal gyrus |
|  | 171 | -62 | -36 | 40 | 3.65 | 0.011 | <0.001 | Left | Supramarginal gyrus |
|  | 133 | 26 | 60 | 20 | 3.61 | 0.013 | <0.001 | Right | Superior frontal gyrus |
|  | 133 | -57 | -52 | -2 | 3.59 | 0.015 | <0.001 | Right | Middle temporal gyrus |
| POST>MALE_POST_ | -- |  |  |  |  |  |  |  |  |
| PERI<MALE_PERI_ | 146 | 36 | 9 | 14 | 4.45 | 0.011 | <0.001 | Right | Insula |
|  | 173 | -18 | 38 | -14 | 4.08 | 0.020 | <0.001 | Left | Orbitofrontal gyrus |
| PERI>MALE_PERI_ | -- |  |  |  |  |  |  |  |  |
| PRE<MALE_PRE_ | 153 | -51 | -28 | 48 | 4.37 | 0.012 | <0.001 | Left | Supramarginal gyrus |
| PRE>MALE_PRE_ | -- |  |  |  |  |  |  |  |  |

*p<0.05, cluster-level corrected, adjusted by age and global activity. Abbreviations: see legend to Supplemental Table S5.

## **Supplementary Table S8**. Cerebral blood flow differences between menopausal groups and age-matched males.

| **Contrast** | **Ke** | **MNI coordinates** | | | **Z** | **P_FWE_*** | **P_uncorr_** | **Side** | **Region** |
| --- | --- | --- | --- | --- | --- | --- | --- | --- | --- |
|  |  | x | y | z |  |  |  |  |  |
| POST<MALE_POST_ | 2237 | 9 | 36 | 0 | 5.80 | <0.001 | <0.001 | Right | Anterior cingulate gyrus |
|  |  | 8 | 38 | -15 | 4.38 |  | <0.001 | Right | Rectal gyrus |
|  |  | 12 | 34 | 18 | 4.03 |  | <0.001 | Right | Anterior cingulate gyrus |
|  | 5773 | 12 | 10 | -10 | 5.01 | <0.001 | <0.001 | Right | Caudate |
|  |  | 18 | 4 | -16 | 4.92 |  | <0.001 | Right | Amygdala |
|  | 516 | -21 | 0 | -16 | 4.08 | 0.003 | <0.001 | Left | Amygdala |
|  |  | -34 | -12 | 3 | 3.97 |  | <0.001 | Left | Putamen |
| POST>MALE_POST_ | 1854 | -48 | 46 | -6 | 4.56 | <0.001 | <0.001 | Left | Inferior frontal gyrus |
|  |  | -40 | 46 | 20 | 4.47 |  | <0.001 | Left | Middle frontal gyrus |
|  | 153 | 40 | 57 | 3 | 4.08 | 0.012 | <0.001 | Right | Middle frontal gyrus |
| PERI<MALE_PERI_ | 8126 | 48 | -20 | 2 | 6.34 | <0.001 | <0.001 | Right | Superior temporal gyrus |
|  |  | 42 | -18 | -14 | 5.72 |  | <0.001 | Right | Hippocampus |
|  |  | 33 | 3 | -2 | 5.46 |  | <0.001 | Right | Putamen |
|  | 8512 | -28 | 12 | 0 | 5.52 | <0.001 | <0.001 | Left | Putamen |
|  |  | -33 | -8 | -16 | 5.48 |  | <0.001 | Left | Hippocampus |
|  | 743 | 20 | -14 | 3 | 4.79 | 0.039 | <0.001 | Right | Thalamus |
|  | 907 | 48 | 14 | 10 | 4.47 | 0.019 | <0.001 | Right | Inferior frontal gyrus |
|  |  | 44 | 27 | 10 | 3.61 |  | <0.001 | Right | Inferior frontal gyrus |
|  | 456 | -21 | -14 | 0 | 4.39 | 0.020 | <0.001 | Left | Putamen |
|  | 1043 | 10 | 35 | 3 | 4.33 | <0.001 | <0.001 | Right | Anterior cingulate gyrus |
|  |  | 8 | 15 | 28 | 4.25 |  | <0.001 | Right | Anterior cingulate gyrus |
| PERI>MALE_PERI_ | 9500 | 14 | -66 | 62 | 4.66 | 0.001 | <0.001 | Right | Superior parietal lobule |
|  |  | -36 | -65 | 55 | 4.99 |  |  | Left | Superior parietal lobule |
| PRE<MALE_PRE_ | 1282 | 50 | -15 | 2 | 5.42 | <0.001 | <0.001 | Right | Superior temporal gyrus |
|  | 96 | 40 | -20 | -12 | 4.07 | 0.010 | <0.001 | Right | Hippocampus |
|  | 216 | 18 | 4 | -16 | 4.06 | 0.005 | <0.001 | Right | Amygdala |
|  | 767 | 12 | 42 | -9 | 3.94 | 0.001 | <0.001 | Right | Orbitofrontal gyrus |
|  |  | 12 | 40 | 10 | 3.71 |  | <0.001 | Right | Anterior cingulate gyrus |
|  | 539 | 33 | -2 | -2 | 3.90 | 0.002 | <0.001 | Right | Putamen |
| PRE>MALE_PRE_ | 561 | 44 | -48 | 54 | 4.97 | 0.003 | <0.001 | Right | Angular gyrus |
|  | 279 | -34 | 40 | 36 | 4.14 | 0.004 | <0.001 | Left | Middle frontal gyrus |
|  | 206 | -32 | -46 | 50 | 3.60 | 0.010 | <0.001 | Left | Superior parietal lobule |

*p<0.05, cluster-level corrected, adjusted by age and global activity.

Abbreviations: see legend to Supplementary Table S5.

## **Supplementary Table S9**. Longitudinal analysis: participants’ characteristics.

|  | **POST** | **MALE_POST_** |
| --- | --- | --- |
| N | 17 | 12 |
| Baseline age, years | 57(3) | 56(4) |
| Time to follow-up, years | 2(2) | 2(2) |
| Education, years | 16(3) | 17(3) |
| Ethnicity, % White | 80 | 82 |
| APOE ε4 carriers, % positive | 49 | 46 |
| Baseline MMSE score | 29(1) | 29(1) |
| Global cognition score, rate (SE) | -0.03(0.06) | 0.01(0.08) |
| Memory score, rate (SE) | -0.03(0.03) | 0.04(0.04) |

Values are means (SD), unless otherwise specified.

Abbreviations: MALE_POST,_ males age-matched to POST; POST, post-menopausal group.

## **Supplementary Table S10**. Two-year gray matter volume changes in post-menopausal women vs. age-matched males.

| **Contrast** | **Ke** | **MNI coordinates** | | | **Z** | **P_FWE_*** | **P_uncorr_** | **Side** | **Region** |
| --- | --- | --- | --- | --- | --- | --- | --- | --- | --- |
|  |  | x | y | z |  |  |  |  |  |
| **POST group** |  |  |  |  |  |  |  |  |  |
| FU<Baseline | 255 | 60 | 24 | 29 | 4.11 | 0.009 | <0.001 | Right | Inferior frontal gyrus |
| FU>Baseline | 240 | 14 | -58 | 30 | 3.56 | 0.017 | <0.001 | Right | Precuneus |
| **MALE_POST_ group** | | | | | | | | | |
| FU<Baseline | *68* | *58* | *-54* | *36* | *2.97* | *0.053* | *<0.001* | *Right* | *Angular gyrus* |
|  | *22* | *51* | *38* | *-14* | *2.74* | *0.084* | *0.003* | *Right* | *Inferior frontal gyrus* |
| FU>Baseline | -- |  |  |  |  |  |  |  |  |

*p<0.05, cluster-level corrected for Family-Type Wise Error (FWE), adjusted by time to follow-up and total intracranial volume. *Clusters showing borderline significance are reported in italics.*

Abbreviations: FU, 2-year follow-up; Ke, cluster extent (voxels); MALE_POST,_ males age-matched to POST; POST, post-menopausal group.

## **Supplementary Table S11**. Two-year FDG glucose metabolism changes in post-menopausal women vs. age-matched males.

| **Contrast** | **Ke** | **MNI coordinates** | | | **Z** | **P*** | **Side** | **Region** |
| --- | --- | --- | --- | --- | --- | --- | --- | --- |
|  |  | x | y | z |  |  |  |  |
| **POST group** |  |  |  |  |  |  |  |  |
| FU<Baseline | 82 | -44 | -45 | 36 | 4.12 | 0.047 | Left | Angular gyrus |
| FU>Baseline | -- |  |  |  |  |  |  |  |
| **MALE_POST_** |  |  |  |  |  |  |  |  |
| FU<Baseline | -- |  |  |  |  |  |  |  |
| FU>Baseline | -- |  |  |  |  |  |  |  |

*p<0.05, cluster-level corrected, adjusted by age, time to follow-up, and global activity.

Abbreviations: see legend to Supplementary Table S10.

## **Supplementary Table S12**. Cognitive measures by age-matched male group comparisons.

|  | **PRE** | **MALE_PRE_** | **PERI** | **MALE_PRE_** | **POST** | **MALE_POST_** |
| --- | --- | --- | --- | --- | --- | --- |
| Global cognition |  |  |  |  |  |  |
| adjusted by age and education | -0.01(0.13) | 0.19(0.14) | 0.02(0.10) | 0.01(0.11) | -0.02(0.09) | -0.14(0.11) |
| adjusted by age, education and APOE-4 status | -0.01(0.14) | 0.20(0.14) | 0.03(0.10) | -0.01(0.11) | -0.02(0.09) | -0.15(0.12) |
| Memory |  |  |  |  |  |  |
| adjusted by age and education | 0.18(0.15) | 0.20(0.15) | 0.06(0.11) | 0.04(0.12) | 0.02(0.09) | -0.09(0.11) |
| adjusted by age, education and APOE-4 status | 0.18(0.15) | 0.20(0.15) | 0.05(0.12) | 0.03(0.12) | 0.02(0.09) | -0.09(0.11) |

Values are means (SE).

Abbreviations: MALE_PRE,_ males age-matched to PRE; MALE_PERI,_ males age-matched to PERI; MALE_POST,_ males age-matched to POST; PERI, peri-menopausal group; POST, post-menopausal group; PRE, pre-menopausal group.

## **Supplementary Table S13**. Correlations between biomarkers and cognition.

| **Biomarker** | **Group** | **Global cognition** | **Memory** | **Global cognition change** | **Memory**  **change** |
| --- | --- | --- | --- | --- | --- |
| Precuneus GMV | POST | 0.149 | 0.311* | -0.078 | 0.586* |
|  | PERI | -0.143 | 0.058 | -- | -- |
|  | PRE | -0.010 | -0.013 | -- | -- |
| Precuneus GMV rate | POST | -- | -- | 0.297 | 0.521* |
| Temporo-parietal ATP/PCr | POST | 0.323* | -0.127 | -- | -- |
|  | PERI | -0.073 | -0.156 | -- | -- |
|  | PRE | 0.232 | 0.141 | -- | -- |
| Temporo-parietal CBF | POST | -0.044 | -0.034 | -- | -- |
|  | PERI | -0.130 | 0.049 | -- | -- |
|  | PRE | 0.121 | 0.359 | -- | -- |

Spearman’s *Rho* coefficients are reported for global cognition and Pearson’s *r*’s for memory scores. *p<0.05, 2-tailed.

Baseline biomarker measures were obtained on all participants specified in Methods. GM volume and cognition rates were obtained only on POST participants. ATP/PCr and CBF measures are the average of temporo-parietal regions. As follow-up CBF and ATP/PCr exams are in process, we did not test for associations between these biomarkers and changes in cognition.

Abbreviations: PERI, peri-menopausal group; POST, post-menopausal group; PRE, pre-menopausal group.
